# Identification and Development of Therapeutics for COVID-19

**Published:** 2021-03-03

**Authors:** Halie M. Rando, Nils Wellhausen, Soumita Ghosh, Alexandra J. Lee, Anna Ada Dattoli, Fengling Hu, James Brian Byrd, Diane N. Rafizadeh, Ronan Lordan, Yanjun Qi, Yuchen Sun, Christian Brueffer, Jeffrey M. Field, Marouen Ben Guebila, Nafisa M. Jadavji, Ashwin N. Skelly, Bharath Ramsundar, Jinhui Wang, Rishi Raj Goel, YoSon Park, COVID-19 Review Consortium, Simina M. Boca, Anthony Gitter, Casey S. Greene

**Affiliations:** Department of Systems Pharmacology and Translational Therapeutics, University of Pennsylvania, Philadelphia, Pennsylvania, United States of America; Department of Biochemistry and Molecular Genetics, University of Colorado School of Medicine, Aurora, Colorado, United States of America; Center for Health AI, University of Colorado School of Medicine, Aurora, Colorado, United States of America · Funded by the Gordon and Betty Moore Foundation (GBMF 4552); Department of Systems Pharmacology and Translational Therapeutics, University of Pennsylvania, Philadelphia, Pennsylvania, United States of America; Institute of Translational Medicine and Therapeutics, Perelman School of Medicine, University of Pennsylvania, Philadelphia, Pennsylvania, United States of America; Department of Systems Pharmacology and Translational Therapeutics, University of Pennsylvania, Philadelphia, Pennsylvania, United States of America · Funded by the Gordon and Betty Moore Foundation (GBMF 4552); Department of Systems Pharmacology & Translational Therapeutics, Perelman School of Medicine, University of Pennsylvania, Philadelphia, PA 19104, USA; Department of Biostatistics, Epidemiology and Informatics, University of Pennsylvania, Philadelphia, Pennsylvania, United States of America; University of Michigan School of Medicine, Ann Arbor, Michigan, United States of America · Funded by NIH K23HL128909; FastGrants; Perelman School of Medicine, University of Pennsylvania, Philadelphia, Pennsylvania, United States of America; Department of Chemistry, University of Pennsylvania, Philadelphia, Pennsylvania, United States of AmericaFunded by NIH Medical Scientist Training Program T32 GM07170; Institute for Translational Medicine and Therapeutics, Perelman School of Medicine, University of Pennsylvania, Philadelphia, PA 19104-5158, USA; Department of Computer Science, University of Virginia, Charlottesville, VA, United States of America; Department of Computer Science, University of Virginia, Charlottesville, VA, United States of America; Department of Clinical Sciences, Lund University, Lund, Sweden; Department of Systems Pharmacology and Translational Therapeutics, Perelman School of Medicine, University of Pennsylvania, Philadelphia, PA 19104, USA; Department of Biostatistics, Harvard School of Public Health, Boston, Massachusetts, United States of America; Biomedical Science, Midwestern University, Glendale, AZ, United States of America; Department of Neuroscience, Carleton University, Ottawa, Ontario, Canada · Funded by the American Heart Association (20AIREA35050015); Perelman School of Medicine, University of Pennsylvania, Philadelphia, Pennsylvania, United States of America; Institute for Immunology, University of Pennsylvania Perelman School of Medicine, Philadelphia, United States of America · Funded by NIH Medical Scientist Training Program T32 GM07170; The DeepChem Project, https://deepchem.io/; Perelman School of Medicine, University of Pennsylvania, Philadelphia, Pennsylvania, United States of America; Institute for Immunology, University of Pennsylvania, Philadelphia, PA, United States of America; Department of Systems Pharmacology and Translational Therapeutics, University of Pennsylvania, Philadelphia, Pennsylvania, United States of America · Funded by NHGRI R01 HG10067; Innovation Center for Biomedical Informatics, Georgetown University Medical Center, Washington, District of Columbia, United States of America; Early Biometrics & Statistical Innovation, Data Science & Artificial Intelligence, R & D, AstraZeneca, Gaithersburg, Maryland, United States of America; Department of Biostatistics and Medical Informatics, University of Wisconsin-Madison, Madison, Wisconsin, United States of America; Morgridge Institute for Research, Madison, Wisconsin, United States of America · Funded by John W. and Jeanne M. Rowe Center for Research in Virology; Department of Systems Pharmacology and Translational Therapeutics, University of Pennsylvania, Philadelphia, Pennsylvania, United States of America; Childhood Cancer Data Lab, Alex’s Lemonade Stand Foundation, Philadelphia, Pennsylvania, United States of America; Department of Biochemistry and Molecular Genetics, University of Colorado School of Medicine, Aurora, Colorado, United States of America; Center for Health AI, University of Colorado School of Medicine, Aurora, Colorado, United States of America · Funded by the Gordon and Betty Moore Foundation (GBMF 4552); the National Human Genome Research Institute (R01 HG010067)

## Abstract

After emerging in China in late 2019, the novel coronavirus SARS-CoV-2 spread worldwide and as of mid-2021 remains a significant threat globally. Only a few coronaviruses are known to infect humans, and only two cause infections similar in severity to SARS-CoV-2: *Severe acute respiratory syndrome-related coronavirus*, a closely related species of SARS-CoV-2 that emerged in 2002, and *Middle East respiratory syndrome-related coronavirus*, which emerged in 2012. Unlike the current pandemic, previous epidemics were controlled rapidly through public health measures, but the body of research investigating severe acute respiratory syndrome and Middle East respiratory syndrome has proven valuable for identifying approaches to treating and preventing novel coronavirus disease 2019 (COVID-19). Building on this research, the medical and scientific communities have responded rapidly to the COVID-19 crisis to identify many candidate therapeutics. The approaches used to identify candidates fall into four main categories: adaptation of clinical approaches to diseases with related pathologies, adaptation based on virological properties, adaptation based on host response, and data-driven identification of candidates based on physical properties or on pharmacological compendia. To date, a small number of therapeutics have already been authorized by regulatory agencies such as the Food and Drug Administration (FDA), while most remain under investigation. The scale of the COVID-19 crisis offers a rare opportunity to collect data on the effects of candidate therapeutics. This information provides insight not only into the management of coronavirus diseases, but also into the relative success of different approaches to identifying candidate therapeutics against an emerging disease.

## 2 Introduction

**Figure 1:**
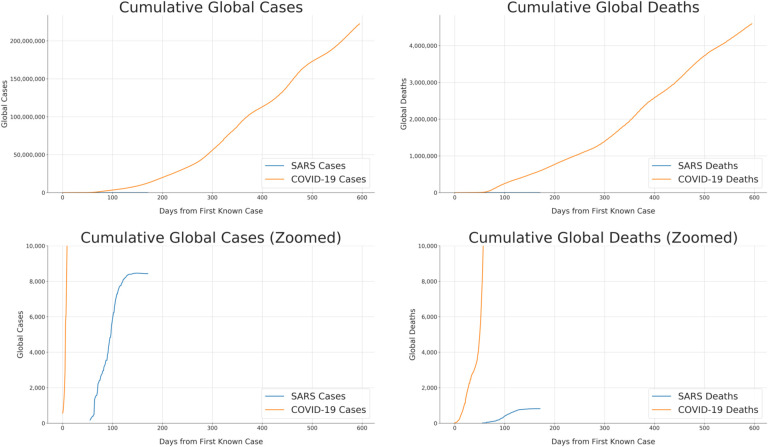
Cumulative global incidence of COVID-19 and SARS. As of September 8, 2021, 222,559,803 COVID-19 cases and 4,596,394 COVID-19 deaths had been reported worldwide since January 22, 2020. A total of 8,432 cases and 813 deaths were reported for SARS from March 17 to July 11, 2003. SARS-CoV-1 was officially contained on July 5, 2003, within 9 months of its appearance [3]. In contrast, SARS-CoV-2 remains a significant global threat nearly two years after its emergence. COVID-19 data are from the COVID-19 Data Repository by the Center for Systems Science and Engineering at Johns Hopkins University [4,5]. SARS data are from the WHO [6] and were obtained from a dataset on GitHub [7]. See https://greenelab.github.io/covid19-review/ for the most recent version of this figure, which is updated daily.

The novel coronavirus *Severe acute respiratory syndrome-related coronavirus 2* (SARS-CoV-2) emerged in late 2019 and quickly precipitated the worldwide spread of novel coronavirus disease 2019 (COVID-19). COVID-19 is associated with symptoms ranging from mild or even asymptomatic to severe, and up to 2% of patients diagnosed with COVID-19 die from COVID-19-related complications such as acute respiratory disease syndrome (ARDS) [1]. As a result, public health efforts have been critical to mitigating the spread of the virus. However, as of mid-2021, COVID-19 remains a significant worldwide concern (Figure 1), with 2021 cases in some regions surging far above the numbers reported during the initial outbreak in early 2020. While a number of vaccines have been developed and approved in different countries starting in late 2020 [2], vaccination efforts have not proceeded at the same pace throughout the world and are not yet close to ending the pandemic.

Due to the continued threat of the virus and the severity of the disease, the identification and development of therapeutic interventions have emerged as significant international priorities. Prior developments during other recent outbreaks of emerging diseases, especially those caused by human coronaviruses (HCoV), have guided biomedical research into the behavior and treatment of this novel coronavirus infection. However, previous emerging HCoV-related disease threats were controlled much more quickly than SARS-CoV-2 through public health efforts (Figure 1). The scale of the COVID-19 pandemic has made the repurposing and development of pharmaceuticals more urgent than in previous coronavirus epidemics.

### 2.1 Lessons from Prior HCoV Outbreaks

At first, SARS-CoV-2’s rapid shift from an unknown virus to a significant worldwide threat closely paralleled the emergence of *Severe acute respiratory syndrome-related coronavirus* (SARS-CoV-1), which was responsible for the 2002–03 SARS epidemic. The first documented case of COVID-19 was reported in Wuhan, China in November 2019, and the disease quickly spread worldwide in the early months of 2020. In comparison, the first case of SARS was reported in November 2002 in the Guangdong Province of China, and it spread within China and then into several countries across continents during the first half of 2003 [3,8,9]. In fact, genome sequencing quickly revealed the virus causing COVID-19 to be a novel betacoronavirus closely related to SARS-CoV-1 [10].

While similarities between these two viruses are unsurprising given their close phylogenetic relationship, there are also some differences in how the viruses affect humans. SARS-CoV-1 infection is severe, with an estimated case fatality rate (CFR) for SARS of 9.5% [8], while estimates of the CFR associated with COVID-19 are much lower, at up to 2% [1]. SARS-CoV-1 is highly contagious and spread primarily by droplet transmission, with a basic reproduction number (R_0_) of 4 (i.e., each person infected was estimated to infect four other people) [8]. There is still some controversy whether SARS-CoV-2 is primarily spread by droplets or is primarily airborne [11,12,13,14]. Most estimates of its R_0_ fall between 2.5 and 3 [1]. Therefore, SARS is thought to be a deadlier and more transmissible disease than COVID-19.

With the 17-year difference between these two outbreaks, there were major differences in the tools available to efforts to organize international responses. At the time that SARS-CoV-1 emerged, no new HCoV had been identified in almost 40 years [9]. The identity of the virus underlying the SARS disease remained unknown until April of 2003, when the SARS-CoV-1 virus was characterized through a worldwide scientific effort spearheaded by the World Health Organization (WHO) [9]. In contrast, the SARS-CoV-2 genomic sequence was released on January 3, 2020 [10], only days after the international community became aware of the novel pneumonia-like illness now known as COVID-19. While SARS-CoV-1 belonged to a distinct lineage from the two other HCoVs known at the time of its discovery [8], SARS-CoV-2 is closely related to SARS-CoV-1 and is a more distant relative of another HCoV characterized in 2012, *Middle East respiratory syndrome-related coronavirus* [15,16]. Significant efforts had been dedicated towards understanding SARS-CoV-1 and MERS-CoV and how they interact with human hosts. Therefore, SARS-CoV-2 emerged under very different circumstances than SARSCoV-1 in terms of scientific knowledge about HCoVs and the tools available to characterize them.

Despite the apparent advantages for responding to SARS-CoV-2 infections, COVID-19 has caused many orders of magnitude more deaths than SARS did (Figure 1). The SARS outbreak was officially determined to be under control in July 2003, with the success credited to infection management practices such as mask wearing [9]. *Middle East respiratory syndrome-related coronavirus* (MERS-CoV) is still circulating and remains a concern; although the fatality rate is very high at almost 35%, the disease is much less easily transmitted, as its R_0_ has been estimated to be 1 [8]. The low R_0_ in combination with public health practices allowed for its spread to be contained [8]. Neither of these trajectories are comparable to that of SARS-CoV-2, which remains a serious threat worldwide over a year and a half after the first cases of COVID-19 emerged (Figure 1).

### 2.2 Potential Approaches to the Treatment of COVID-19

Therapeutic interventions can utilize two approaches: they can either mitigate the effects of an infection that harms an infected person, or they can hinder the spread of infection within a host by disrupting the viral life cycle. The goal of the former strategy is to reduce the severity and risks of an active infection, while for the latter, it is to inhibit the replication of a virus once an individual is infected, potentially freezing disease progression. Additionally, two major approaches can be used to identify interventions that might be relevant to managing an emerging disease or a novel virus: drug repurposing and drug development. Drug repurposing involves identifying an existing compound that may provide benefits in the context of interest [17]. This strategy can focus on either approved or investigational drugs, for which there may be applicable preclinical or safety information [17]. Drug development, on the other hand, provides an opportunity to identify or develop a compound specifically relevant to a particular need, but it is often a lengthy and expensive process characterized by repeated failure [18]. Drug repurposing therefore tends to be emphasized in a situation like the COVID-19 pandemic due to the potential for a more rapid response.

**Figure 2:**
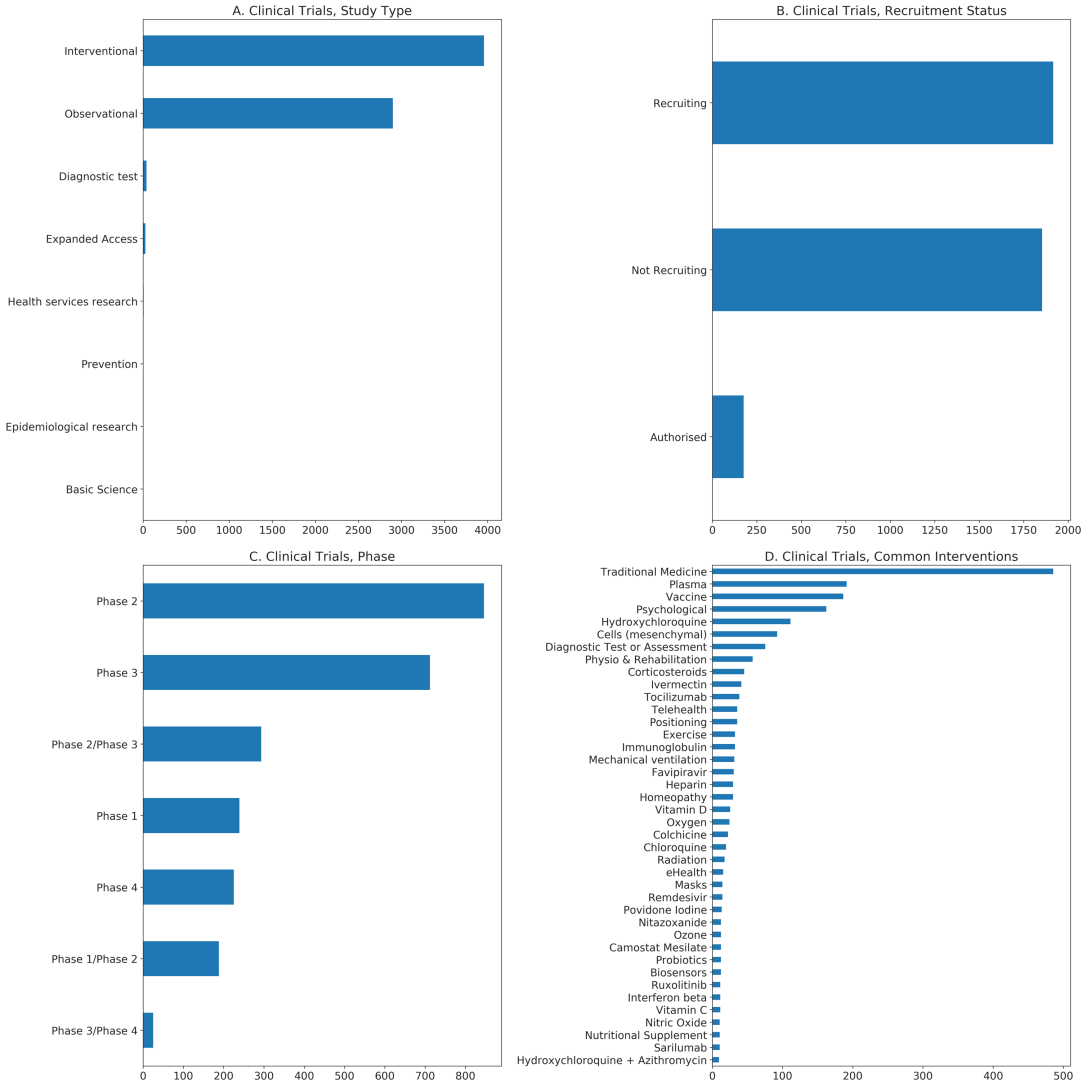
COVID-19 clinical trials. Trials data are from the University of Oxford Evidence-Based Medicine Data Lab’s COVID-19 TrialsTracker [19]. As of December 31, 2020, there were 6,987 COVID-19 clinical trials of which 3,962 were interventional. The study types include only types used in at least five trials. Only interventional trials are analyzed in the figures depicting status, phase, and intervention. Of the interventional trials, 98 trials had reported results as of December 31, 2020. Recruitment status and trial phase are shown only for interventional trials in which the status or phase is recorded. Common interventions refers to interventions used in at least ten trials. Combinations of interventions, such as hydroxychloroquine with azithromycin, are tallied separately from the individual interventions. See https://greenelab.github.io/covid19-review/ for the most recent version of this figure, which is updated daily.

Even from the early months of the pandemic, studies began releasing results from analyses of approved and investigational drugs in the context of COVID-19. The rapid timescale of this response meant that, initially, most evidence came from observational studies, which compare groups of patients who did and did not receive a treatment to determine whether it may have had an effect. This type of study can be conducted rapidly but is subject to confounding. In contrast, randomized controlled trials (RCTs) are the gold-standard method for assessing the effects of an intervention. Here, patients are prospectively and randomly assigned to treatment or control conditions, allowing for much stronger interpretations to be drawn; however, data from these trials take much longer to collect. Both approaches have proven to be important sources of information in the development of a rapid response to the COVID-19 crisis, but as the pandemic draws on and more results become available from RCTs, more definitive answers are becoming available about proposed therapeutics. Interventional clinical trials are currently investigating or have investigated a large number of possible therapeutics and combinations of therapeutics for the treatment of COVID-19 (Figure 2).

The purpose of this review is to provide an evolving resource tracking the status of efforts to repurpose and develop drugs for the treatment of COVID-19. We highlight four strategies that provide different paradigms for the identification of potential pharmaceutical treatments. The WHO guidelines [20] and a systematic review [21] are complementary living documents that summarize COVID-19 therapeutics.

## 3 Repurposing Drugs for Symptom Management

A variety of symptom profiles with a range of severity are associated with COVID-19 [1]. In many cases, COVID-19 is not life threatening. A study of COVID-19 patients in a hospital in Berlin, Germany reported that the highest risk of death was associated with infection-related symptoms, such as sepsis, respiratory symptoms such as ARDS, and cardiovascular failure or pulmonary embolism [22]. Similarly, an analysis in Wuhan, China reported that respiratory failure (associated with ARDS) and sepsis/multi-organ failure accounted for 69.5% and 28.0% of deaths, respectively, among 82 deceased patients [23]. COVID-19 is characterized by two phases. The first is the acute response, where an adaptive immune response to the virus is established and in many cases can mitigate viral damage to organs [24]. The second phase characterizes more severe cases of COVID-19. Here, patients experience a cytokine storm, whereby excessive production of cytokines floods into circulation, leading to systemic inflammation, immune dysregulation, and multiorgan dysfunction that can cause multiorgan failure and death if untreated [25]. ARDS-associated respiratory failure can occur during this phase. Cytokine dysregulation was also identified in patients with SARS [26,27].

In the early days of the COVID-19 pandemic, physicians sought to identify potential treatments that could benefit patients, and in some cases shared their experiences and advice with the medical community on social media sites such as Twitter [28]. These on-the-ground treatment strategies could later be analyzed retrospectively in observational studies or investigated in an interventional paradigm through RCTs. Several notable cases involved the use of small-molecule drugs, which are synthesized compounds of low molecular weight, typically less than 1 kilodalton (kDa) [29]. Small-molecule pharmaceutical agents have been a backbone of drug development since the discovery of penicillin in the early twentieth century [30]. It and other antibiotics have long been among the best known applications of small molecules to therapeutics, but biotechnological developments such as the prediction of protein-protein interactions (PPIs) have facilitated advances in precise targeting of specific structures using small molecules [30]. Small molecule drugs today encompass a wide range of therapeutics beyond antibiotics, including antivirals, protein inhibitors, and many broad-spectrum pharmaceuticals.

Many treatments considered for COVID-19 have relied on a broad-spectrum approach. These treatments do not specifically target a virus or particular host receptor, but rather induce broad shifts in host biology that are hypothesized to be potential inhibitors of the virus. This approach relies on the fact that when a virus enters a host, the host becomes the virus’s environment. Therefore, the state of the host can also influence the virus’s ability to replicate and spread. The administration and assessment of broad-spectrum small-molecule drugs on a rapid time course was feasible because they are often either available in hospitals, or in some cases may also be prescribed to a large number of out-patients. One of the other advantages is that these well-established compounds, if found to be beneficial, are often widely available, in contrast to boutique experimental drugs.

In some cases, prior data was available from experiments examining the response of other HCoVs or HCoV infections to a candidate drug. In addition to non-pharmaceutical interventions such as encouraging non-intubated patients to adopt a prone position [31], knowledge about interactions the large-scale study of clinical applications than the COVID-19 pandemic is. As a result, COVID-19 has presented the first opportunity to robustly evaluate treatments that were common during prior HCoV outbreaks to determine their clinical efficacy. The first year of the COVID-19 pandemic demonstrated that there are several different trajectories that these clinically suggested, widely available candidates can follow when assessed against a widespread, novel viral threat.

One approach to identifying candidate small molecule drugs was to look at the approaches used to treat SARS and MERS. Treatment of SARS and MERS patients prioritized supportive care and symptom management [8]. Among the clinical treatments for SARS and MERS that were explored, there was generally a lack of evidence indicating whether they were effective. Most of the supportive treatments for SARS were found inconclusive in meta-analysis [32], and a 2004 review reported that not enough evidence was available to make conclusions about most treatments [33]. However, one strategy adopted from prior HCoV outbreaks is currently the best-known treatment for severe cases of COVID-19. Corticosteroids represent broad-spectrum treatments and are a well-known, widely available treatment for pneumonia [34,35,36,37,38,39] that have also been debated as a possible treatment for ARDS [40,41,42,43,44,45]. Corticosteroids were also used and subsequently evaluated as possible supportive care for SARS and MERS. In general, studies and meta-analyses did not identify support for corticosteroids to prevent mortality in these HCoV infections [46,47,48]; however, one found that the effects might be masked by variability in treatment protocols, such as dosage and timing [33]. While the corticosteroids most often used to treat SARS were methylprednisolone and hydrocortisone, availability issues for these drugs at the time led to dexamethasone also being used in North America [49].

Dexamethasone (9α-fluoro-16α-methylprednisolone) is a synthetic corticosteroid that binds to glucocorticoid receptors [50,51]. It functions as an anti-inflammatory agent by binding to glucocorticoid receptors with higher affinity than endogenous cortisol [52]. Dexamethasone and other steroids are widely available and affordable, and they are often used to treat community-acquired pneumonia [53] as well as chronic inflammatory conditions such as asthma, allergies, and rheumatoid arthritis [54,55,56]. Immunosuppressive drugs such as steroids are typically contraindicated in the setting of infection [57], but because COVID-19 results in hyperinflammation that appears to contribute to mortality via lung damage, immunosuppression may be a helpful approach to treatment [58]. A clinical trial that began in 2012 recently reported that dexamethasone may improve outcomes for patients with ARDS [40], but a meta-analysis of a small amount of available data about dexamethasone as a treatment for SARS suggested that it may, in fact, be associated with patient harm [59]. However, the findings in SARS may have been biased by the fact that all of the studies examined were observational and a large number of inconclusive studies were not included [60]. The questions of whether and when to counter hyperinflammation with immunosuppression in the setting of COVID-19 (as in SARS [27]) was an area of intense debate, as the risks of inhibiting antiviral immunity needed to be weighed against the beneficial anti-inflammatory effects [61]. As a result, guidelines early in the pandemic typically recommended avoiding treating COVID-19 patients with corticosteroids such as dexamethasone [59].

Despite this initial concern, dexamethasone was evaluated as a potential treatment for COVID-19 (Appendix 1). Dexamethasone treatment comprised one arm of the multi-site Randomized Evaluation of COVID-19 Therapy (RECOVERY) trial in the United Kingdom [62]. This study found that the 28-day mortality rate was lower in patients receiving dexamethasone than in those receiving standard of care (SOC). However, this finding was driven by differences in mortality among patients who were receiving mechanical ventilation or supplementary oxygen at the start of the study. The report indicated that dexamethasone reduced 28-day mortality relative to SOC in patients who were ventilated (29.3% versus 41.4%) and among those who were receiving oxygen supplementation (23.3% versus 26.2%) at randomization, but not in patients who were breathing independently (17.8% versus 14.0%). These findings also suggested that dexamethasone may have reduced progression to mechanical ventilation, especially among patients who were receiving oxygen support at randomization. Other analyses have supported the importance of disease course in determining the efficacy of dexamethasone: additional results suggest greater potential for patients who have experienced symptoms for at least seven days and patients who were not breathing independently [63]. A meta-analysis that evaluated the results of the RECOVERY trial alongside trials of other corticosteroids, such as hydrocortisone, similarly concluded that corticosteroids may be beneficial to patients with severe COVID-19 who are receiving oxygen supplementation [64]. Thus, it seems likely that dexamethasone is useful for treating inflammation associated with immunopathy or cytokine release syndrome (CRS), which is a condition caused by detrimental overactivation of the immune system [1]. In fact, corticosteroids such as dexamethasone are sometimes used to treat CRS [65]. Guidelines were quickly updated to encourage the use of dexamethasone in severe cases [66], and this affordable and widely available treatment rapidly became a valuable tool against COVID-19 [67], with demand surging within days of the preprint’s release [68].

## 4 Approaches Targeting the Virus

**Figure 3:**
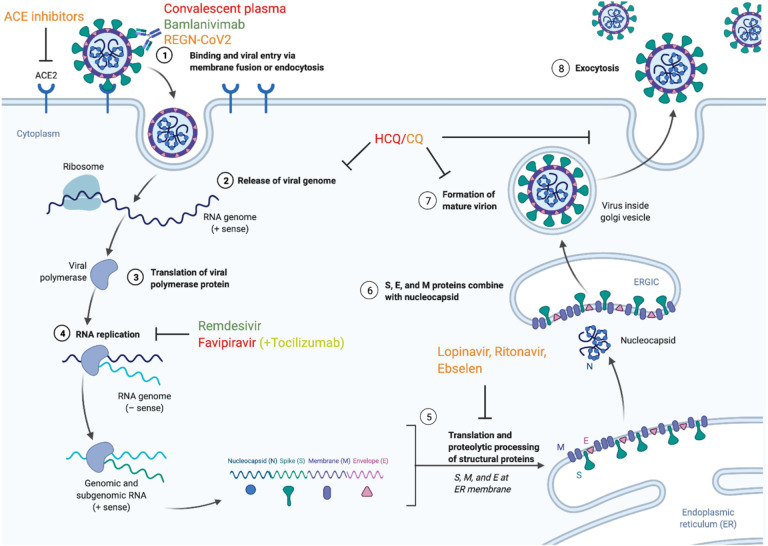
Mechanisms of Action for Potential Therapeutics Potential therapeutics currently being studied can target the SARS-CoV-2 virus or modify the host environment through many different mechanisms. Here, the relationships between the virus, host cells, and several therapeutics are visualized. Drug names are color-coded according to the grade assigned to them by the Center for Cytokine Storm Treatment & Laboratory’s CORONA Project [70] (Green = A, Lime = B, Orange = C, and Red = D).

Therapeutics that directly target the virus itself hold the potential to prevent people infected with SARS-CoV-2 from developing potentially damaging symptoms (Figure 3). Such drugs typically fall into the broad category of antivirals. Antiviral therapies hinder the spread of a virus within the host, rather than destroying existing copies of the virus, and these drugs can vary in their specificity to a narrow or broad range of viral targets. This process requires inhibiting the replication cycle of a virus by disrupting one of six fundamental steps [69]. In the first of these steps, the virus attaches to and enters the host cell through endocytosis. Then the virus undergoes uncoating, which is classically defined as the release of viral contents into the host cell. Next, the viral genetic material enters the nucleus where it gets replicated during the biosynthesis stage. During the assembly stage, viral proteins are translated, allowing new viral particles to be assembled. In the final step new viruses are released into the extracellular environment. Although antivirals are designed to target a virus, they can also impact other processes in the host and may have unintended effects. Therefore, these therapeutics must be evaluated for both efficacy and safety. As the technology to respond to emerging viral threats has also evolved over the past two decades, a number of candidate treatments have been identified for prior viruses that may be relevant to the treatment of COVID-19.

Many antiviral drugs are designed to inhibit the replication of viral genetic material during the biosynthesis step. Unlike DNA viruses, which can use the host enzymes to propagate themselves, RNA viruses like SARS-CoV-2 depend on their own polymerase, the RNA-dependent RNA polymerase (RdRP), for replication [71,72]. RdRP is therefore a potential target for antivirals against RNA viruses. Disruption of RdRP is the proposed mechanism underlying the treatment of SARS and MERS with ribavirin [73]. Ribavirin is an antiviral drug effective against other viral infections that was often used in combination with corticosteroids and sometimes interferon (IFN) medications to treat SARS and MERS [9]. However, analyses of its effects in retrospective and *in vitro* analyses of SARS and the SARS-CoV-1 virus, respectively, have been inconclusive [9]. While IFNs and ribavirin have shown promise in *in vitro* analyses of MERS, their clinical effectiveness remains unknown [9]. The current COVID-19 pandemic has provided an opportunity to assess the clinical effects of these treatments. As one example, ribivarin was also used in the early days of COVID-19, but a retrospective cohort study comparing patients who did and did not receive ribivarin revealed no effect on the mortality rate [74].

Since nucleotides and nucleosides are the natural building blocks for RNA synthesis, an alternative approach has been to explore nucleoside and nucleotide analogs for their potential to inhibit viral replication. Analogs containing modifications to nucleotides or nucleosides can disrupt key processes including replication [75]. A single incorporation does not influence RNA transcription; however, multiple events of incorporation lead to the arrest of RNA synthesis [76]. One candidate antiviral considered for the treatment of COVID-19 is favipiravir (Avigan), also known as T-705, which was discovered by Toyama Chemical Co., Ltd. [77]. It was previously found to be effective at blocking viral amplification in several influenza subtypes as well as other RNA viruses, such as *Flaviviridae* and *Picornaviridae*, through a reduction in plaque formation [78] and viral replication in Madin-Darby canine kidney cells [79]. Favipiravir (6-fluoro-3-hydroxy-2-pyrazinecarboxamide) acts as a purine and purine nucleoside analogue that inhibits viral RNA polymerase in a dose-dependent manner across a range of RNA viruses, including influenza viruses [80,81,82,83,84]. Biochemical experiments showed that favipiravir was recognized as a purine nucleoside analogue and incorporated into the viral RNA template. In 2014, the drug was approved in Japan for the treatment of influenza that was resistant to conventional treatments like neuraminidase inhibitors [85]. Though initial analyses of favipiravir in observational studies of its effects on COVID-19 patients were promising, recent results of two small RCTs suggest that it is unlikely to affect COVID-19 outcomes (Appendix 1).

In contrast, another nucleoside analog, remdesivir, is one of the few treatments against COVID-19 that has received FDA approval. Remdesivir (GS-5734) is an intravenous antiviral that was proposed by Gilead Sciences as a possible treatment for Ebola virus disease. It is metabolized to GS-441524, an adenosine analog that inhibits a broad range of polymerases and then evades exonuclease repair, causing chain termination [86,87,88]. Gilead received an emergency use authorization (EUA) for remdesivir from the FDA early in the pandemic (May 2020) and was later found to reduce mortality and recovery time in a double-blind, placebo-controlled, phase III clinical trial performed at 60 trial sites, 45 of which were in the United States [89,90,91,92]. Subsequently, the WHO Solidarity trial, a large-scale, open-label trial enrolling 11,330 adult in-patients at 405 hospitals in 30 countries around the world, reported no effect of remdesivir on in-hospital mortality, duration of hospitalization, or progression to mechanical ventilation [93]. Therefore, additional clinical trials of remdesivir in different patient pools and in combination with other therapies may be needed to refine its use in the clinic and determine the forces driving these differing results. Remdesivir offers proof of principle that SARS-CoV-2 can be targeted at the level of viral replication, since remdesivir targets the viral RNA polymerase at high potency. Identification of such candidates depends on knowledge about the virological properties of a novel threat. However, the success and relative lack of success, respectively, of remdesivir and favipiravir underscore the fact that drugs with similar mechanisms will not always produce similar results in clinical trials.

## 5 Disrupting Host-Virus Interactions

### 5.1 Interrupting Viral Colonization of Cells

Some of the most widely publicized examples of efforts to repurpose drugs for COVID-19 are broad-spectrum, small-molecule drugs where the mechanism of action made it seem that the drug might disrupt interactions between SARS-CoV-2 and human host cells (Figure 3). However, the exact outcomes of such treatments are difficult to predict *a priori*, and there are several examples where early enthusiasm was not borne out in subsequent trials. One of the most famous examples of an analysis of whether a well-known medication could provide benefits to COVID-19 patients came from the assessment of chloroquine (CQ) and hydroxychloroquine (HCQ), which are used for the treatment and prophylaxis of malaria as well as the treatment of lupus erythematosus and rheumatoid arthritis in adults [94]. These drugs are lysosomotropic agents, meaning they are weak bases that can pass through the plasma membrane. It was thought that they might provide benefits against SARS-CoV-2 by interfering with the digestion of antigens within the lysosome and inhibiting CD4 T-cell stimulation while promoting the stimulation of CD8 T-cells [95]. These compounds also have anti-inflammatory properties [95] and can decrease the production of certain key cytokines involved in the immune response, including interleukin-6 (IL-6) and inhibit the stimulation of Toll-like receptors (TLR) and TLR signaling [95].

*In vitro* analyses reported that CQ inhibited cell entry of SARS-CoV-1 [96] and that both CQ and HCQ inhibited viral replication within cultured cells [97], leading to early hope that it might provide similar therapeutic or protective effects in patients. However, while the first publication on the clinical application of these compounds to the inpatient treatment of COVID-19 was very positive [98], it was quickly discredited [99]. Over the following months, extensive evidence emerged demonstrating that CQ and HCQ offered no benefits for COVID-19 patients and, in fact, carried the risk of dangerous side effects (Appendix 1). The nail in the coffin came when findings from the large-scale RECOVERY trial were released on October 8, 2020. This study enrolled 11,197 hospitalized patients whose physicians believed it would not harm them to participate and used a randomized, open-label design to study the effects of HCQ compared to standard of care (SOC) at 176 hospitals in the United Kingdom [100]. Rates of COVID-19-related mortality did not differ between the control and HCQ arms, but patients receiving HCQ were slightly more likely to die due to cardiac events. Patients who received HCQ also had a longer duration of hospitalization than patients receiving usual care and were more likely to progress to mechanical ventilation or death (as a combined outcome). As a result, enrollment in the HCQ arm of the RECOVERY trial was terminated early [101]. The story of CQ/HCQ therefore illustrates how initial promising *in vitro* analyses can fail to translate to clinical usefulness.

A similar story has arisen with the broad-spectrum, small-molecule anthelmintic ivermectin, which is a synthetic analog of avermectin, a bioactive compound produced by a microorganism known as *Streptomyces avermectinius* and *Streptomyces avermitilis* [102,103]. Avermectin disrupts the ability of parasites to avoid the host immune response by blocking glutamate-gated chloride ion channels in the peripheral nervous system from closing, leading to hyperpolarization of neuronal membranes, disruption of neural transmission, and paralysis [102,104,105]. Ivermectin has been used since the early 1980s to treat endo- and ecto-parasitic infections by helminths, insects, and arachnids in veterinary contexts [102,106] and since the late 1980s to treat human parasitic infections as well [102,104]. More recent research has indicated that ivermectin might function as a broad-spectrum antiviral by disrupting the trafficking of viral proteins by both RNA and DNA viruses [105,107,108], although most of these studies have demonstrated this effect *in vitro* [108]. The potential for antiviral effects on SARSCoV-2 were investigated *in vitro*, and ivermectin was found to inhibit viral replication in a cell line derived from Vero cells (Vero-hSLAM) [109]. However, inhibition of viral replication was achieved at concentrations that were much higher than that explored by existing dosage guidelines [110,111], which are likely to be associated with significant side effects due to the increased potential that the compound could cross the mammalian blood-brain barrier [112,113].

Retrospective studies and small RCTs began investigating the effects of standard doses of this low-cost, widely available drug. One retrospective study reported that ivermectin reduced all-cause mortality [114] while another reported no difference in clinical outcomes or viral clearance [115]. Small RCTs enrolling less than 50 patients per arm have also reported a wide array of positive [116,117,118,119,120] and negative results [121,122]. A slightly larger RCT enrolling 115 patients in two arms reported inconclusive results [123]. Hope for the potential of ivermectin peaked with the release of a preprint reporting results of a multicenter, double-blind RCT where a four-day course of ivermectin was associated with clinical improvement and earlier viral clearance in 400 symptomatic patients and 200 close contacts [124]; however, concerns were raised about both the integrity of the data and the paper itself [125,126], and this study was removed by the preprint server Research Square [127]. A similarly sized RCT suggested no effect on the duration of symptoms among 400 patients split evenly across the intervention and control arms [128], and although meta-analyses have reported both null [129,130] and beneficial [131,132,133,134,135,136,137,138] effects of ivermectin on COVID-19 outcomes, the certainty is likely to be low [132]. These findings are potentially biased by a small number of low-quality studies, including the preprint that has been taken down [139], and the authors of one [140] have issued a notice [131] that they will revise their study with the withdrawn study removed. Thus, much like HCQ/CQ, enthusiasm for research that either has not or should not have passed peer review has led to large numbers of patients worldwide receiving treatments that might not have any effect or could even be harmful. Additionally, comments on the now-removed preprint include inquiries into how best to self-administer veterinary ivermectin as a prophylactic [127], and the FDA has posted information explaining why veterinary ivermectin should not be taken by humans concerned about COVID-19 [141]. Ivermectin is now one of several candidate therapeutics being investigated in the large-scale TOGETHER [142] and PRINCIPLE [143] clinical trials. The TOGETHER trial, which previously demonstrated no effect of HCQ and lopinavir-ritonavir [144], released preliminary results in early August 2021 suggesting that ivermectin also has no effect on COVID-19 outcomes [145].

While CQ/HCQ and ivermectin are well-known medications that have long been prescribed in certain contexts, investigation of another well-established type of pharmaceutical was facilitated by the fact that it was already being taken by a large number of COVID-19 patients. Angiotensin-converting enzyme inhibitors (ACEIs) and angiotensin II receptor blockers (ARBs) are among today’s most commonly prescribed medications, often being used to control blood pressure [146,147]. In the United States, for example, they are prescribed well over 100,000,000 times annually [148]. Prior to the COVID-19 pandemic, the relationship between ACE2, ACEIs, and SARS had been considered as possible evidence that ACE2 could serve as a therapeutic target [149], and the connection had been explored through *in vitro* and molecular docking analysis [150] but ultimately was not pursued clinically [151]. Data from some animal models suggest that several, but not all, ACEIs and several ARBs increase ACE2 expression in the cells of some organs [152], but clinical studies have not established whether plasma ACE2 expression is increased in humans treated with these medications [153]. In this case, rather than introducing ARBs/ACEIs, a number of analyses have investigated whether discontinuing use affects COVID-19 outcomes. An initial observational study of the association of exposure to ACEIs or ARBs with outcomes in COVID-19 was retracted from the *New England Journal of Medicine* [154] due to concerns related to data availability [155]. As RCTs have become available, they have demonstrated no effect of continuing versus discontinuing ARBs/ACEIs on patient outcomes [156,157] (Appendix 1). Thus, once again, despite a potential mechanistic association with the pathology of SARS-CoV-2 infection, these medications were not found to influence the trajectory of COVID-19 illness.

For medications that are widely known and common, clinical research into their efficacy against a novel threat can be developed very quickly. This feasibility can present a double-edged sword. For example, HCQ and CQ were incorporated into SOC in many countries early in the pandemic and had to be discontinued once their potential to harm COVID-19 patients became apparent [158,159]. Dexamethasone remains the major success story from this category of repurposed drugs and is likely to have saved a large number of lives since summer 2020 [67].

### 5.2 Manipulating the Host Immune Response

Treatments based on understanding a virus and/or how a virus interacts with the human immune system can fall into two categories: they can interact with the innate immune response, which is likely to be a similar response across viruses, or they can be specifically designed to imitate the adaptive immune response to a particular virus. In the latter case, conservation of structure or behavior across viruses enables exploring whether drugs developed for one virus can treat another. During the COVID-19 pandemic, a number of candidate therapeutics have been explored in these categories, with varied success.

Knowledge gained from trying to understand SARS-CoV-1 and MERS-CoV from a fundamental biological perspective and characterize how they interact with the human immune system provides a theoretical basis for identifying candidate therapies. Biologics are a particularly important class of drugs for efforts to address HCoV through this paradigm. They are produced from components of living organisms or viruses, historically primarily from animal tissues [160]. Biologics have become increasingly feasible to produce as recombinant DNA technologies have advanced [160].

There are many differences on the development side between biologics and synthesized pharmaceuticals, such as small molecule drugs. Typically, biologics are orders of magnitude larger than small molecule drugs and are catabolized by the body to their amino acid components [161]. They are often heat sensitive, and their toxicity can vary, as it is not directly associated with the primary effects of the drug; in general, their physiochemical properties are much less understood compared to small molecules [161]. Biologics include significant medical breakthroughs such as insulin for the management of diabetes and vaccines, as well monoclonal antibodies (mAbs) and interferons (IFNs), which can be used to target the host immune response after infection.

mAbs have revolutionized the way we treat human diseases and have become some of the best-selling drugs in the pharmaceutical market in recent years [162]. There are currently 79 FDA approved mAbs on the market, including antibodies for viral infections (e.g. Ibalizumab for *Human immunodeficiency virus* and Palivizumab for *Respiratory syncytial virus*) [162,163]. Virus-specific neutralizing antibodies commonly target viral surface glycoproteins or host structures, thereby inhibiting viral entry through receptor binding interference [164,165]. This interference is predicted to reduce the viral load, mitigate disease, and reduce overall hospitalization. mAbs can be designed for a particular virus, and significant advances have been made in the speed at which new mAbs can be identified and produced. At the time of the SARS and MERS epidemics, interest in mAbs to reduce infection was never realized [166,167], but this allowed for mAbs to quickly be considered among the top candidates against COVID-19.

#### 5.2.1 Biologics and the Innate Immune Response

Deaths from COVID-19 often occur when inflammation becomes dysregulated following an immune response to the SARS-CoV-2 virus. Therefore, one potential approach to reducing COVID-19 mortality rates is to manage the inflammatory response in severely ill patients. One candidate therapeutic identified that uses this mechanism is tocilizumab (TCZ). TCZ is a mAb that was developed to manage chronic inflammation caused by the continuous synthesis of the cytokine IL-6 [168]. IL-6 is a pro-inflammatory cytokine belonging to the interleukin family, which is comprised by immune system regulators that are primarily responsible for immune cell differentiation. Often used to treat chronic inflammatory conditions such as rheumatoid arthritis [168], TCZ has become a pharmaceutical of interest for the treatment of COVID-19 because of the role IL-6 plays in this disease. It has also been approved to treat CRS caused by CAR-T treatments [169]. While the secretion of IL-6 can be associated with chronic conditions, IL-6 is a key player in the innate immune response and is secreted by macrophages in response to the detection of pathogen-associated molecular patterns and damage-associated molecular patterns [168]. An analysis of 191 in-patients at two Wuhan hospitals revealed that blood concentrations of IL-6 differed between patients who did and did not recover from COVID-19. Patients who ultimately died had higher IL-6 levels at admission than those who recovered [170]. Additionally, IL-6 levels remained higher throughout the course of hospitalization in the patients who ultimately died [170].

Currently, TCZ is being administered either as a monotherapy or in combination with other treatments in 73 interventional COVID-19 clinical trials (Figure 2). A number of retrospective studies have been conducted in several countries [171,172,173,174,175,176]. In general, these studies have reported a positive effect of TCZ on reducing mortality in COVID-19 patients, although due to their retrospective designs, significant limitations are present in all of them (Appendix 1). It was not until February 11, 2021 that a preprint describing preliminary results of the first RCT of TCZ was released as part of the RECOVERY trial [177]. TCZ was found to reduce 28-day mortality from 33% in patients receiving SOC alone to 29% in those receiving TCZ. Combined analysis of the RECOVERY trial data with data from smaller RCTs suggested a 13% reduction in 28-day mortality [177]. While this initial report did not include the full results expected from the RECOVERY trial, this large-scale, RCT provides strong evidence that TCZ may offer benefits for COVID-19 patients. The RECOVERY trial along with results from several other RCTs [178,179,180,181,182] were cited as support for the EUA issued for TCZ in June 2021 [183]. However, the fact that TCZ suppresses the immune response means that it does carry risks for patients, especially a potential risk of secondary infection (Appendix 1).

TCZ is just one example of a candidate drug targeting the host immune response and specifically excessive inflammation. For example, interferons (IFNs) have also been investigated; these are a family of cytokines critical to activating the innate immune response against viral infections. Synairgen has been investigating a candidate drug, SNG001, which is an IFN-*β*-1a formulation to be delivered to the lungs via inhalation [184] that they reported reduced progression to ventilation in a double-blind, placebo-controlled, multi-center study of 101 patients with an average age in the late 50s [185,186]. However, these findings were not supported by the large-scale WHO Solidarity trial, which reported no significant effect of IFN-β−1a on patient survival during hospitalization [93], although differences in the designs of the two studies, and specifically the severity of illness among enrolled patients, may have influenced their divergent outcomes (Appendix 1). Other biologics influencing inflammation are also being explored (Appendix 1). It is also important that studies focused on inflammation as a possible therapeutic target consider the potential differences in baseline inflammation among patients from different backgrounds, which may be caused by differing life experiences (see [187]).

#### 5.2.2 Biologics and the Adaptive Immune Response

While TCZ is an example of an mAb focused on managing the innate immune response, other treatments are more specific, targeting the adaptive immune response after an infection. In some cases, treatments can utilize biologics obtained directly from recovered individuals. From the very early days of the COVID-19 pandemic, polyclonal antibodies from convalescent plasma were investigated as a potential treatment for COVID-19 [188,189]. Convalescent plasma was used in prior epidemics including SARS, Ebola Virus Disease, and even the 1918 Spanish Influenza [188,190]. Use of convalescent plasma transfusion (CPT) over more than a century has aimed to reduce symptoms and improve mortality in infected people [190], possibly by accelerating viral clearance [188]. However, it seems unlikely that this classic treatment confers any benefit for COVID-19 patients. Several systematic reviews have investigated whether CPT reduced mortality in COVID-19 patients, and although findings from early in the pandemic (up to April 19, 2020) did support use of CPT [190], the tide has shifted as the body of available literature has grown [191]. While titer levels were suggested as a possible determining factor in the success of CPT against COVID-19 [192], the large-scale RECOVERY trial evaluated the effect of administering high-titer plasma specifically and found no effect on mortality or hospital discharge over a 28-day period [193]. These results thus suggest that, despite initial optimism and an EUA from the FDA, CPT is unlikely to be an effective therapeutic for COVID-19.

A different narrative is shaping up around the use of mAbs specifically targeting SARS-CoV-2. During the first SARS epidemic in 2002, neutralizing antibodies (nAbs) were found in SARSCoV-1-infected patients [194,195]. Several studies following up on these findings identified various S-glycoprotein epitopes as the major targets of nAbs against SARS-CoV-1 [196]. Coronaviruses use trimeric spike (S) glycoproteins on their surface to bind to the host cell, allowing for cell entry [197,198]. Each S glycoprotein protomer is comprised of an S1 domain, also called the receptor binding domain (RBD), and an S2 domain. The S1 domain binds to the host cell while the S2 domain facilitates the fusion between the viral envelope and host cell membranes [196]. The genomic identity between the RBD of SARS-CoV-1 and SARS-CoV-2 is around 74% [199]. Due to this high degree of similarity, preexisting antibodies against SARSCoV-1 were initially considered candidates for neutralizing activity against SARS-CoV-2. While some antibodies developed against the SARS-CoV-1 spike protein showed cross-neutralization activity with SARS-CoV-2 [200,201], others failed to bind to SARS-CoV-2 spike protein at relevant concentrations [202]. Cross-neutralizing activities were dependent on whether the epitope recognized by the antibodies were conserved between SARS-CoV-1 and SARS-CoV-2 [200].

Technological advances in antibody drug design as well as in structural biology massively accelerated the discovery of novel antibody candidates and the mechanisms by which they interact with the target structure. Within just a year of the structure of the SARS-CoV-2 spike protein being published, an impressive pipeline of monoclonal antibodies targeting SARS-CoV-2 entered clinical trials, with hundreds more candidates in preclinical stages. The first human monoclonal neutralizing antibody specifically against the SARS-CoV-2 S glycoprotein was developed using hybridoma technology [203], where antibody-producing B-cells developed by mice are inserted into myeloma cells to produce a hybrid cell line (the hybridoma) that is grown in culture. The 47D11 antibody clone was able to cross-neutralize SARS-CoV-1 and SARS-CoV-2. This antibody (now ABVV-47D11) has recently entered clinical trials in collaboration with AbbVie. Additionally, an extensive monoclonal neutralizing antibody pipeline has been developed to combat the ongoing pandemic, with over 50 different antibodies in clinical trials [204]. Thus far, the monotherapy sotrovimab and two antibody cocktails (bamlanivimab/estesevimab and casirivimab/imdevimab) have been granted EUAs by the FDA.

One of the studied antibody cocktails consists of bamlanivimab and estesevimab. Bamlanivimab (Ly-CoV555) is a human mAb that was derived from convalescent plasma donated by a recovered COVID-19 patient, evaluated in research by the National Institute of Allergy and Infectious Diseases (NIAID), and subsequently developed by AbCellera and Eli Lilly. The neutralizing activity of bamlanivimab was initially demonstrated *in vivo* using a nonhuman primate model [205]. Based on these positive preclinical data, Eli Lilly initiated the first human clinical trial for a monoclonal antibody against SARS-CoV-2. The phase 1 trial, which was conducted in hospitalized COVID-19 patients, was completed in August 2020 [206]. Estesevimab (LY-CoV016 or JS-016) is also a monoclonal neutralizing antibody against the spike protein of SARS-CoV-2. It was initially developed by Junshi Biosciences and later licensed and developed through Eli Lilly. A phase 1 clinical trial to assess the safety of etesevimab was completed in October 2020 [207]. Etesevimab was shown to bind a different epitope on the spike protein than bamlanivimab, suggesting that the two antibodies used as a combination therapy would further enhance their clinical use compared to a monotherapy [208]. To assess the efficacy and safety of bamlanivimab alone or in combination with etesevimab for the treatment of COVID-19, a phase 2/3 trial (BLAZE-1) [209] was initiated. The interim analysis of the phase 2 portion suggested that bamlanivimab alone was able to accelerate the reduction in viral load [210]. However, more recent data suggests that only the bamlanivimab/etesevimab combination therapy is able to reduce viral load in COVID-19 patients [208]. Based on this data, the combination therapy received an EUA for COVID-19 from the FDA in February 2021 [211].

A second therapy is comprised of casirivimab and imdevimab (REGN-COV2). Casirivimab (REGN10933) and imdevimab (REGN10987) are two monoclonal antibodies against the SARSCoV-2 spike protein. They were both developed by Regeneron in a parallel high-throughput screening (HTS) to identify neutralizing antibodies from either humanized mice or patient-derived convalescent plasma [212]. In these efforts, multiple antibodies were characterized for their ability to bind and neutralize the SARS-CoV-2 spike protein. The investigators hypothesized that an antibody cocktail, rather than each individual antibody, could increase the therapeutic efficacy while minimizing the risk for virus escape. Therefore, the authors tested pairs of individual antibodies for their ability to simultaneously bind the RBD of the spike protein. Based on this data, casirivimab and imdevimab were identified as the lead antibody pair, resulting in the initiation of two clinical trials [213,214]. Data from this phase 1–3 trial published in the *New England Journal of Medicine* shows that the REGN-COV2 antibody cocktail reduced viral load, particularly in patients with high viral load or whose endogenous immune response had not yet been initiated [215]. However, in patients who already initiated an immune response, exogenous addition of REGN-COV2 did not improve the endogenous immune response. Both doses were well tolerated with no serious events related to the antibody cocktail. Based on this data, the FDA granted an EUA for REGN-COV2 in patients with mild to moderate COVID-19 who are at risk of developing severe disease [216]. Ongoing efforts are trying to evaluate the efficacy of REGN-COV2 to improve clinical outcomes in hospitalized patients [213].

Sotrovimab is the most recent mAb to receive an EUA. It was identified in the memory B cells of a 2003 survivor of SARS [217] and was found to be cross-reactive with SARS-CoV-2 [201]. This cross-reactivity is likely attributable to conservation within the epitope, with 17 out of 22 residues conserved between the two viruses, four conservatively substituted, and one semi-conservatively substituted [201]. In fact, these residues are highly conserved among sarbecoviruses, a clade that includes SARS-CoV-1 and SARS-CoV-2 [201]. This versatility has led to it being characterized as a “super-antibody” [218], a potent, broadly neutralizing antibody [219]. Interim analysis of data from a clinical trial [220] reported high safety and efficacy of this mAb in 583 COVID-19 patients [221]. Compared to placebo, sotrovimab was found to be 85% more effective in reducing progression to the primary endpoint, which was the proportion of patients who, within 29 days, were either hospitalized for more than 24 hours or died. Additionally, rates of adverse events were comparable, and in some cases lower, among patients receiving sotrovimab compared to patients receiving a placebo. Sotrovimab therefore represents a mAb therapeutic that is effective against SARS-CoV-2 and may also be effective against other sarbecoviruses.

Several potential limitations remain in the application of mAbs to the treatment of COVID-19. One of the biggest challenges is identifying antibodies that not only bind to their target, but also prove to be beneficial for disease management. Currently, use of mAbs is limited to people with mild to moderate disease that are not hospitalized, and it has yet to be determined whether they can be used as a successful treatment option for severe COVID-19 patients. While preventing people from developing severe illness provides significant benefits, patients with severe illness are at the greatest risk of death, and therefore therapeutics that provide benefits against severe illness are particularly desirable. It remains to be seen whether mAbs confer any benefits for patients in this category.

Another concern about therapeutics designed to amplify the response to a specific viral target is that they may need to be modified as the virus evolves. With the ongoing global spread of new SARS-CoV-2 variants, there is a growing concern that mutations in SARS-CoV-2 spike protein could escape antibody neutralization, thereby reducing the efficacy of monoclonal antibody therapeutics and vaccines. A comprehensive mutagenesis screen recently identified several amino acid substitutions in the SARS-CoV-2 spike protein that can prevent antibody neutralization [222]. While some mutations result in resistance to only one antibody, others confer broad resistance to multiple mAbs as well as polyclonal human sera, suggesting that some amino acids are “hotspots” for antibody resistance. However, it was not investigated whether the resistance mutations identified result in a fitness advantage. Accordingly, an impact on neutralizing efficiency has been reported for the B.1.1.7 (Alpha) variant first identified in the UK and the B.1.351 (Beta) variant first identified in in South Africa [223,224,225]. As of June 25, 2021, the CDC recommended a pause in the use of bamlanivimab and etesevimab due to decreased efficacy against the P.1 (Gamma) and B.1.351 (Beta) variants of SARS-CoV-2 [226]. While the reported impact on antibody neutralization needs to be confirmed *in vivo*, it suggests that some adjustments to therapeutic antibody treatments may be necessary to maintain the efficacy that was reported in previous clinical trials.

Several strategies have been employed to try to mitigate the risk of diminished antibody neutralization. Antibody cocktails such as those already holding an EUA may help overcome the risk for attenuation of the neutralizing activity of a single monoclonal antibody. These cocktails consist of antibodies that recognize different epitopes on the spike protein, decreasing the likelihood that a single amino acid change can cause resistance to all antibodies in the cocktail. However, neutralizing resistance can emerge even against an antibody cocktail if the individual antibodies target subdominant epitopes [224]. Another strategy is to develop broadly neutralizing antibodies that target structures that are highly conserved, as these are less likely to mutate [227,228] or to target epitopes that are insensitive to mutations [229]. Sotrovimab, one such “super-antibody”, is thought to be somewhat robust to neutralization escape [230] and has been found to be effective against all variants assessed as of August 12, 2021 [231]. Another antibody (ADG-2) targets a highly conserved epitope that overlaps the hACE2 binding site of all clade 1 sarbecoviruses [232]. Prophylactic administration of ADG-2 in an immunocompetent mouse model of COVID-19 resulted in protection against viral replication in the lungs and respiratory burden. Since the epitope targeted by ADG-2 represents an Achilles’ heel for clade 1 sarbecoviruses, this antibody, like sotrovimab, might be a promising candidate against all circulating variants as well as emerging SARS-related coronaviruses. To date, it has fared well against the Alpha, Beta, Gamma, and Delta variants [231].

The development of mAbs against SARS-CoV-2 has made it clear that this technology is rapidly adaptable and offers great potential for the response to emerging viral threats. However, additional investigation may be needed to adapt mAb treatments to SARS-CoV-2 as it evolves and potentially to pursue designs that confer benefits for patients at the greatest risk of death. While polyclonal antibodies from convalescent plasma have been evaluated as a treatment for COVID-19, these studies have suggested fewer potential benefits against SARS-CoV-2 than mAbs; convalescent plasma therapy has been thoroughly reviewed elsewhere [188,189]. Thus, advances in biologics for COVID-19 illustrate that an understanding of how the host and virus interact can guide therapeutic approaches. The FDA authorization of two combination mAb therapies, in particular, underscores the potential for this strategy to allow for a rapid response to a novel pathogen. Additionally, while TCZ is not yet as established, this therapy suggests that the strategy of using biologics to counteract the cytokine storm response may provide therapies for the highest-risk patients.

## 6 High-Throughput Screening for Drug Repurposing

The drug development process is slow and costly, and developing compounds specifically targeted to an emerging viral threat is not a practical short-term solution. Screening existing drug compounds for alternative indications is a popular alternative [233,234,235,236]. HTS has been a goal of pharmaceutical development since at least the mid-1980s [237]. Traditionally, phenotypic screens were used to test which compounds would induce a desired change in *in vitro* or *in vivo* models, focusing on empirical, function-oriented exploration naïve to molecular mechanism [238,239,240]. In many cases, these screens utilize large libraries that encompass a diverse set of agents varying in many pharmacologically relevant properties (e.g., [241]). The compounds inducing a desired effect could then be followed up on. Around the turn of the millennium, advances in molecular biology allowed for HTS to shift towards screening for compounds interacting with a specific molecular target under the hypothesis that modulating that target would have a desired effect. These approaches both offer pros and cons, and today a popular view is that they are most effective in combination [238,240,242].

Today, some efforts to screen compounds for potential repurposing opportunities are experimental, but others use computational HTS approaches [233,243]. Computational drug repurposing screens can take advantage of big data in biology [17] and as a result are much more feasible today than during the height of the SARS and MERS outbreaks in the early 2000s and early 2010s, respectively. Advancements in robotics also facilitate the experimental component of HTS [235]. For viral diseases, the goal of drug repurposing is typically to identify existing drugs that have an antiviral effect likely to impede the virus of interest. While both small molecules and biologics can be candidates for repurposing, the significantly lower price of many small molecule drugs means that they are typically more appealing candidates [244].

Depending on the study design, screens vary in how closely they are tied to a hypothesis. As with the candidate therapeutics described above, high-throughput experimental or computational screens can proceed based on a hypothesis. Just as remdesivir was selected as a candidate antiviral because it is a nucleoside analog [245], so too can high-throughput screens select libraries of compounds based on a molecular hypothesis. Likewise, when the library of drugs is selected without basis in a potential mechanism, a screen can be considered hypothesis free [245]. Today, both types of analyses are common both experimentally and computationally. Both strategies have been applied to identifying candidate therapeutics against SARS-CoV-2.

### 6.1 Hypothesis-Driven Screening

Hypothesis-driven screens often select drugs likely to interact with specific viral or host targets or drugs with desired clinical effects, such as immunosuppressants. There are several properties that might identify a compound as a candidate for an emerging viral disease. Drugs that interact with a target that is shared between pathogens (i.e., a viral protease or a polymerase) or between a viral pathogen and another illness (i.e., a cancer drug with antiviral potential) are potential candidates, as are drugs that are thought to interact with additional molecular targets beyond those they were developed for [243]. Such research can be driven by *in vitro* or *in silico* experimentation. Computational analyses depend on identifying compounds that modulate pre-selected proteins in the virus or host. As a result, they build on experimental research characterizing the molecular features of the virus, host, and candidate compounds [236].

One example of the application of this approach to COVID-19 research comes from work on protease inhibitors. Studies have shown that viral proteases play an important role in the life cycle of viruses, including coronaviruses, by modulating the cleavage of viral polyprotein precursors [246]. Several FDA-approved drugs target proteases, such as lopinavir and ritonavir for HIV infection and simeprevir for hepatitis C virus infection. Serine protease inhibitors were previously suggested as possible treatments for SARS and MERS [247]. One early study [197] suggested that camostat mesylate, a protease inhibitor, could block the entry of SARS-CoV-2 into lung cells *in vitro*. Two polyproteins encoded by the SARS-CoV-2 replicase gene, pp1a and pp1ab, are critical for viral replication and transcription [248]. These polyproteins must undergo proteolytic processing, which is usually conducted by main protease (M^Pro^), a 33.8-kDa SARSCoV-2 protease that is therefore fundamental to viral replication and transcription. Therefore, it was hypothesized that compounds targeting M^Pro^ could be used to prevent or slow the replication of the SARS-CoV-2 virus.

Both computational and experimental approaches facilitated the identification of compounds that might inhibit SARS-CoV-2 M^Pro^. In 2005, computer-aided design facilitated the development of a Michael acceptor inhibitor, now known as N3, to target M^Pro^ of SARS-like coronaviruses [249]. N3 binds in the substrate binding pocket of M^Pro^ in several HCoV [249,250,251,252]. The structure of N3-bound SARS-CoV-2 M^Pro^ has been solved, confirming the computational prediction that N3 would similarly bind in the substrate binding pocket of SARS-CoV-2 [248]. N3 was tested *in vitro* on SARS-CoV-2-infected Vero cells, which belong to a line of cells established from the kidney epithelial cells of an African green monkey, and was found to inhibit SARS-CoV-2 [248]. A library of approximately 10,000 compounds was screened in a fluorescence resonance energy transfer assay constructed using SARS-CoV-2 M^Pro^ expressed in *Escherichia coli* [248].

Six leads were identified in this hypothesis-driven screen. *In vitro* analysis revealed that ebselen had the strongest potency in reducing the viral load in SARS-CoV-2-infected Vero cells [248]. Ebselen is an organoselenium compound with anti-inflammatory and antioxidant properties [253]. Molecular dynamics analysis further demonstrated the potential for ebselen to bind to M^Pro^ and disrupt the protease’s enzymatic functions [254]. However, ebselen is likely to be a promiscuous binder, which could diminish its therapeutic potential [248,255], and compounds with higher specificity may be needed to translate this mechanism effectively to clinical trials. In July 2020, phase II clinical trials commenced to assess the effects of SPI-1005, an investigational drug from Sound Pharmaceuticals that contains ebselen [256], on 60 adults presenting with each of moderate [257] and severe [258] COVID-19. Other M^Pro^ inhibitors are also being evaluated in clinical trials [259,260,260]. Pending the results of clinical trials, N3 remains a computationally interesting compound based on both computational and experimental data, but whether these potential effects will translate to the clinic remains unknown.

### 6.2 Hypothesis-Free Screening

Hypothesis-free screens use a discovery-driven approach, where screens are not targeted to specific viral proteins, host proteins, or desired clinical modulation. Hypothesis-free drug screening began twenty years ago with the testing of libraries of drugs experimentally. Today, like many other areas of biology, *in silico* analyses have become increasingly popular and feasible through advances in biological big data [245,261]. Many efforts have collected data about interactions between drugs and SARS-CoV-2 and about the host genomic response to SARS-CoV-2 exposure, allowing for hypothesis-free computational screens that seek to identify new candidate therapeutics. Thus, they utilize a systems biology paradigm to extrapolate the effect of a drug against a virus based on the host interactions with both the virus and the drug [236].

Resources such as the COVID-19 Drug and Gene Set Library, which at the time of its publication contained 1,620 drugs sourced from 173 experimental and computational drug sets and 18,676 human genes sourced from 444 gene sets [262], facilitate such discovery-driven approaches. Analysis of these databases indicated that some drugs had been identified as candidates across multiple independent analyses, including high-profile candidates such as CQ/HCQ and remdesivir [262]. Computational screening efforts can then mine databases and other resources to identify potential PPIs among the host, the virus, and established and/or experimental drugs [263]. Subject matter expertise from human users may be integrated to varying extents depending on the platform (e.g,. [263,264]). These resources have allowed studies to identify potential therapeutics for COVID-19 without an *a priori* reason for selecting them.

One example of a hypothesis-free screen for COVID-19 drugs comes from a PPI-network-based analysis that was published early in the pandemic [265]. Here, researchers cloned the proteins expressed by SARS-CoV-2 *in vitro* and quantified 332 viral-host PPI using affinity purification mass spectrometry [265]. They identified two SARS-CoV-2 proteins (Nsp6 and Orf9c) that interacted with host Sigma-1 and Sigma-2 receptors. Sigma receptors are located in the endoplasmic reticulum of many cell types, and type 1 and 2 Sigma receptors have overlapping but distinct affinities for a variety of ligands [266]. Molecules interacting with the Sigma receptors were then analyzed and found to have an effect on viral infectivity *in vitro* [265]. A follow-up study evaluated the effect of perturbing these 332 proteins in two cells lines, A549 and Caco-2, using knockdown and knockout methods, respectively, and found that the replication of SARSCoV-2 in cells from both lines was dependent on the expression of *SIGMAR1*, which is the gene that encodes the Sigma-1 receptor [267]. Following these results, drugs interacting with Sigma receptors were suggested as candidates for repurposing for COVID-19 (e.g., [268]). Because many well-known and affordable drugs interact with the Sigma receptors [265,269], they became a major focus of drug repurposing efforts. Some of the drugs suggested by the apparent success of Sigma receptor-targeting drugs were already being investigated at the time. HCQ, for example, forms ligands with both Sigma-1 and Sigma-2 receptors and was already being explored as a candidate therapeutic for COVID-19 [265]. Thus, this computational approach yielded interest in drugs whose antiviral activity was supported by initial *in vitro* analyses.

Follow-up research, however, called into question whether the emphasis on drugs interacting with Sigma receptors might be based on a spurious association [270]. This study built on the prior work by examining whether antiviral activity among compounds correlated with their affinity for the Sigma receptors and found that it did not. The study further demonstrated that cationic amphiphilicity was a shared property among many of the candidate drugs identified through both computational and phenotypic screens and that it was likely to be the source of many compounds’ proposed antiviral activity [270]. Cationic amphiphilicity is associated with the induction of phospholipidosis, which is when phospholipids accumulate in the lysosome [271]. Phospholipidosis can disrupt viral replication by inhibiting lipid processing [272] (see discussion of HCQ in Appendix 1). However, phospholipidosis is known to translate poorly from *in vitro* models to *in vivo* models or clinical applications. Thus, this finding suggested that these screens were identifying compounds that shared a physiochemical property rather than a specific target [270]. The authors further demonstrated that antiviral activity against SARS-CoV-2 *in vitro* was correlated with the induction of phospholipidosis for drugs both with and without cationic amphiphilicity [270]. This finding supports the idea that the property of cationic amphicility was being detected as a proxy for the shared effect of phospholipidosis [270]. They demonstrated that phospholipidosis-inducing drugs were not effective at preventing viral propagation *in vivo* in a murine model of COVID-19 [270]. Therefore, removing hits that induce phospholipidosis from computational and *in vitro* experimental repurposing screens (e.g., [273]) may help emphasize those that are more likely to provide clinical benefits. This work illustrates the importance of considering confounding variables in computational screens, a principle that has been incorporated into more traditional approaches to drug development [274].

One drug that acts on Sigma receptors does, however, remain a candidate for the treatment of COVID-19. Several psychotropic drugs target Sigma receptors in the central nervous system and thus attracted interest as potential COVID-19 therapeutics following the findings of two host-virus PPI studies [275]. For several of these drugs, the *in vitro* antiviral activity [267] was not correlated with their affinity for the Sigma-1 receptor [270,275] but was correlated with phospholipidosis [270]. However, fluvoxamine, a selective serotonin reuptake inhibitor that is a particularly potent Sigma-1 receptor agonist [275], has shown promise as a preventative of severe COVID-19 in a preliminary analysis of data from the large-scale TOGETHER trial [145]. As of August 6, 2021, this trial had collected data from over 1,400 patients in the fluvoxamine arm of their study, half of whom received a placebo [145]. Only 74 patients in the fluvoxamine group had progressed to hospitalization for COVID-19 compared to 107 in the placebo group, corresponding to a relative risk of 0.69; additionally, the relative risk of mortality between the two groups was calculated at 0.71. These findings support the results of small clinical trials that have found fluvoxamine to reduce clinical deterioration relative to a placebo [276,277]. However, the ongoing therapeutic potential of fluvoxamine does not contradict the finding that hypothesis-free screening hits can be driven by confounding factors. The authors point out that its relevance would not just be antiviral as it has a potential immunomodulatory mechanism [276]. It has been found to be protective against septic shock in an *in vivo* mouse model [278]. It is possible that fluvoxamine also exerts an antiviral effect [279]. Thus, Sigma-1 receptor activity may contribute to fluvoxamine’s potential effects in treating COVID-19, but is not the only mechanism by which this drug can interfere with disease progression.

### 6.3 Potential and Limitations of High-Throughput Analyses

Computational screening allows for a large number of compounds to be evaluated to identify those most likely to display a desired behavior or function. This approach can be guided by a hypothesis or can aim to discover underlying characteristics that produce new hypotheses about the relationship between a host, a virus, and candidate pharmaceuticals. The examples outlined above illustrate that HTS-based evaluations of drug repurposing can potentially provide valuable insights. Computational techniques were used to design compounds targeting M^Pro^ based on an understanding of how this protease aids viral replication, and M^Pro^ inhibitors remain promising candidates [235], although the clinical trial data is not yet available. Similarly, computational analysis correctly identified the Sigma-1 receptor as a protein of interest. Although the process of identifying which drugs might modulate the interaction led to an emphasis on candidates that ultimately have not been supported, fluvoxamine remains an appealing candidate. The difference between the preliminary evidence for fluvoxamine compared to other drugs that interact with Sigma receptors underscores a major critique of hypothesis-free HTS in particular: while these approaches allow for brute force comparison of a large number of compounds against a virus of interest, they lose the element of expertise that is associated with most successes in drug repurposing [245].

There are also practical limitations to these methods. One concern is that computational analyses inherently depend on the quality of the data being evaluated. The urgency of the COVID-19 pandemic led many research groups to pivot towards computational HTS research without familiarity with best practices in this area [235]. As a result, there is an excessive amount of information available from computational studies [280], but not all of it is high-quality. Additionally, the literature used to identify and validate targets can be difficult to reproduce [281], which may pose challenges to target-based experimental screening and to *in silico* screens. Some efforts to repurpose antivirals have focused on host, rather than viral, proteins [236], which might be expected to translate poorly *in vivo* if the targeted proteins serve essential functions in the host. Concerns about the practicality of hypothesis-free screens to gain novel insights are underscored by the fact that very few or possibly no success stories have emerged from hypothesis-free screens over the past twenty years [245]. These findings suggest that data-driven research can be an important component of the drug repurposing ecosystem, but that drug repurposing efforts that proceed without a hypothesis, an emphasis on biological mechanisms, or an understanding of confounding effects may not produce viable candidates.

## 7 Considerations in Balancing Different Approaches

The approaches described here offer a variety of advantages and limitations in responding to a novel viral threat and building on existing bodies of knowledge in different ways. Medicine, pharmacology, basic science (especially virology and immunology), and biological data science can all provide different insights and perspectives for addressing the challenging question of which existing drugs might provide benefits against an emerging viral threat. A symptom management-driven approach allows clinicians to apply experience with related diseases or related symptoms to organize a rapid response aimed at saving the lives of patients already infected with a new disease. Oftentimes, the pharmaceutical agents that are applied are small-molecule, broad-spectrum pharmaceuticals that are widely available and affordable to produce, and they may already be available for other purposes, allowing clinicians to administer them to patients quickly either with an EUA or off-label. In this vein, dexamethasone has emerged as the strongest treatment against severe COVID-19 (Table 1).

Alternatively, many efforts to repurpose drugs for COVID-19 have built on information gained through basic scientific research of HCoV. Understanding how related viruses function has allowed researchers to identify possible pharmacological strategies to disrupt pathogenesis (Figure 3). Some of the compounds identified through these methods include small-molecule antivirals, which can be boutique and experimental medications like remdesivir (Table 1). Other candidate drugs that intercept host-pathogen interactions include biologics, which imitate the function of endogenous host compounds. Most notably, several mAbs that have been developed (casirivimab, imdevimab, bamlanivimab and etesevimab) or repurposed (sotrovimab, tocilizumab) have now been granted EUAs (Table 1). Although not discussed here, several vaccine development programs have also met huge success using a range of strategies [2].

All of the small-molecule drugs evaluated and most of the biologics are repurposed, and thus hinge on a theoretical understanding of how the virus interacts with a human host and how pharmaceuticals can be used to modify those interactions rather than being designed specifically against SARS-CoV-2 or COVID-19. As a result, significant attention has been paid to computational approaches that automate the identification of potentially desirable interactions. However, work in COVID-19 has made it clear that relevant compounds can also be masked by confounds, and spurious associations can drive investment in candidate therapeutics that are unlikely to translate to the clinic. Such spurious hits are especially likely to impact hypothesis-free screens. However, hypothesis-free screens may still be able to contribute to the drug discovery or repurposing ecosystem, assuming the computational arm of HTS follows the same trends seen in its experimental arm. In 2011, a landmark study in drug discovery demonstrated that although more new drugs were discovered using target-based rather than phenotypic approaches, the majority of drugs with a novel molecular mechanism of action (MMOA) were identified in phenotypic screens [282]. This pattern applied only to first-in-class drugs, with most follower drugs produced by target-based screening [239]. These findings suggest that target-based drug discovery is more successful when building on a known MMOA, and that modulating a target is most valuable when the target is part of a valuable MMOA [240]. Building on this, many within the field suggested that mechanism-informed phenotypic investigations may be the most useful approach to drug discovery [238,240,242]. As it stands, data-driven efforts to identify patterns in the results of computational screens allowed researchers to notice the shared property of cationic amphicility among many of the hits from computational screening analyses [270]. While easier said than done, efforts to fill in the black box underlying computational HTS and recognize patterns among the identified compounds aid in moving data-oriented drug repurposing efforts in this direction.

The unpredictable nature of success and failure in drug repurposing for COVID-19 thus highlights one of the tenets of phenotypic screening: there are a lot of “unknown unknowns”, and a promising mechanism at the level of an MMOA will not necessarily propagate up to the pathway, cellular, or organismal level [238]. Despite the fact that apparently mechanistically relevant drugs may exist, identifying effective treatments for a new viral disease is extremely challenging. Targets of repurposed drugs are often non-specific, meaning that the MMOA can appear to be relevant to COVID-19 without a therapeutic or prophylactic effect being observed in clinical trials. The difference in the current status of remdesivir and favipiravir as treatments for COVID-19 (Table 1) underscores how difficult to predict whether a specific compound will produce a desired effect, even when the mechanisms are similar. Furthermore, the fact that many candidate COVID-19 therapeutics were ultimately identified because of their shared propensity to induce phospholipidosis underscores how challenging it can be to identify a mechanism *in silico* or *in vitro* that will translate to a successful treatment. While significant progress has been made thus far in the pandemic, the therapeutic landscape is likely to continue to evolve as more results become available from clinical trials and as efforts to develop novel therapeutics for COVID-19 progress.

## 8 Towards the Next HCoV Threat

Only very limited testing of candidate therapies was feasible during the SARS and MERS epidemics, and as a result, few treatments were available at the outset of the COVID-19 pandemic. Even corticosteroids, which were used to treat SARS patients, were a controversial therapeutic prior to the release of the results of the large RECOVERY trial. The scale and duration of the COVID-19 pandemic has made it possible to conduct large, rigorous RCTs such as RECOVERY, Solidarity, TOGETHER, and others. As results from these trials have continued to emerge, it has become clear that small clinical trials often produce spurious results. In the case of HCQ/CQ, the therapeutic had already attracted so much attention based on small, preliminary (and in some cases, methodologically concerning) studies that it took the results of multiple large studies before attention began to be redirected to more promising candidates [283]. In fact, most COVID-19 clinical trials lack the statistical power to reliably test their hypotheses [284,285]. In the face of an urgent crisis like COVID-19, the desire to act quickly is understandable, but it is imperative that studies maintain strict standards of scientific rigor [235,274], especially given the potential dangers of politicization, as illustrated by HCQ/CQ [286]. Potential innovations in clinical trial structure, such as adaptable clinical trials with master protocols [287] or the sharing of data among small clinical trials [285] may help to address future crises and to bolster the results from smaller studies, respectively.

In the long-term, new drugs specific for treatment of COVID-19 may also enter development. Development of novel drugs is likely to be guided by what is known about the pathogenesis and molecular structure of SARS-CoV-2. For example, understanding the various structural components of SARS-CoV-2 may allow for the development of small molecule inhibitors of those components. Crystal structures of the SARS-CoV-2 main protease have been resolved [248,288]. Much work remains to be done to determine further crystal structures of other viral components, understand the relative utility of targeting different viral components, perform additional small molecule inhibitor screens, and determine the safety and efficacy of the potential inhibitors. While still nascent, work in this area is promising. Over the longer term, this approach and others may lead to the development of novel therapeutics specifically for COVID-19 and SARS-CoV-2. Such efforts are likely to prove valuable in managing future emergent HCoV, just as research from the SARS and MERS pandemic has provided a basis for the COVID-19 response.

## Figures and Tables

**Table 1:** Summary table of candidate therapeutics examined in this manuscript. “Grade” is the rating given to each treatment by the Systematic Tracker of Off-label/Repurposed Medicines Grades (STORM) maintained by the Center for Cytokine Storm Treatment & Laboratory (CSTL) at the University of Pennsylvania [70]. A grade of A indicates that a treatment is considered effective, B that all or most RCTs have shown positive results, C that RCT data are not yet available, and D that multiple RCTs have produced negative results. Treatments not in the STORM database are indicated as N/A. FDA status is also provided where available. The evidence available is based on the progression of the therapeutic through the pharmaceutical development pipeline, with RCTs as the most informative source of evidence. The effectiveness is summarized based on the current available evidence; large trials such as RECOVERY and Solidarity are weighted heavily in this summary. This table was last updated on August 20, 2021.

Treatment	Grade	Category	FDA Status	Evidence Available	Suggested Effectiveness
Dexamethasone	A	Small molecule, broad spectrum	Used off-label	RCT	Supported: RCT shows improved outcomes over SOC, especially in severe cases such as CRS
Remdesivir	A	Small molecule, antiviral, adenosine analog	Approved for COVID-19 (and EUA for combination with baricitinib)	RCT	Mixed: Conflicting evidence from large WHO-led Solidarity trial vs US-focused RCT and other studies
Tocilizumab	A	Biologic, monoclonal antibody	EUA	RCT	Mixed: It appears that TCZ may work well in combination with dexamethasone in severe cases, but not as monotherapy
Sotrovimab	N/A	Biological, monoclonal antibody	EUA	RCT	Supported: Phase 2/3 clinical trial showed reduced hospitalization/death
Bamlanivimab and etesevimab	B & N/A	Biologic, monoclonal antibodies	EUA	RCT	Supported: Phase 2 clinical trial showed reduction in viral load, but FDA pause recommended because may be less effective against Delta variant
Casirivimab and imdevimab	N/A	Biologic, monoclonal antibodies	EUA	RCT	Supported: Reduced viral load at interim analysis
Fluvoxamine	B	Smallmolecule, Sigma-1 receptor agonist	N/A	RCT	Supported: Support from two small RCTs and preliminary support from interim analysis of TOGETHER
SNG001	B	Biologic, interferon	None	RCT	Mixed: Support from initial RCT but no effect found in WHO’s Solidarity trial
M^Pro^ Protease Inhibitors	N/A	Small molecule, protease inhibitor	None	Computational prediction, *in vitro* studies	Unknown
ARBs & ACEIs	C	Small molecule, broad spectrum	None	Observational studies and some RCTs	Not supported: Observational study retracted, RCTs suggest no association
Favipiravir	D	Small molecule, antiviral, nucleoside analog	None	RCT	Not supported: RCTs do not show significant improvements for individuals taking this treatment, good safety profile
HCQ/CQ	D	Small molecule, broad spectrum	None	RCT	Not supported, possibly harmful: Non-blinded RCTs showed no improvement over SOC, safety profile may be problematic
Convalescent plasma transfusion	D	Biologic, polyclonal antibodies	EUA	RCT	Mixed: Supported in small trials but not in large-scale RECOVERY trial
Ivermectin	D	Small molecule, broad spectrum	None	RCT	Mixed: Mixed results from small RCTs, major supporting RCT now withdrawn, preliminary results of large RCT (TOGETHER) suggest no effect on emergency room visits or hospitalization for COVID-19

## 1.2 Importance

The COVID-19 pandemic is a rapidly evolving crisis. With the worldwide scientific community shifting focus onto the SARS-CoV-2 virus and COVID-19, a large number of possible pharmaceutical approaches for treatment and prevention have been proposed. What was known about each of these potential interventions evolved rapidly throughout 2020 and 2021. This fast-paced area of research provides important insight into how the ongoing pandemic can be managed and also demonstrates the power of interdisciplinary collaboration to rapidly understand a virus and match its characteristics with existing or novel pharmaceuticals. As illustrated by the continued threat of viral epidemics during the current millennium, a rapid and strategic response to emerging viral threats can save lives. In this review, we explore how different modes of identifying candidate therapeutics have borne out during COVID-19.

## 1 Appendix: Identification and Development of Therapeutics for COVID-19

### 1.1 Dexamethasone

In order to understand how dexamethasone reduces inflammation, it is necessary to consider the stress response broadly. In response to stress, corticotropin releasing hormone stimulates the release of neurotransmitters known as catecholamines, such as epinephrine, and steroid hormones known as glucocorticoids, such as cortisol [289,290]. While catecholamines are often associated with the fight-or-flight response, the specific role that glucocorticoids play is less clear, although they are thought to be important to restoring homeostasis [291]. Immune challenge is a stressor that is known to interact closely with the stress response. The immune system can therefore interact with the central nervous system; for example, macrophages can both respond to and produce catecholamines [289]. Additionally, the production of both catecholamines and glucocorticoids is associated with inhibition of proinflammatory cytokines such as IL-6, IL-12, and tumor necrosis factor-α (TNF α) and the stimulation of anti-inflammatory cytokines such as IL-10, meaning that the stress response can regulate inflammatory immune activity [290]. Administration of dexamethasone has been found to correspond to dose-dependent inhibition of IL-12 production, but not to affect IL-10 [292]; the fact that this relationship could be disrupted by administration of a glucocorticoid-receptor antagonist suggests that it is regulated by the receptor itself [292]. Thus, the administration of dexamethasone for COVID-19 is likely to simulate the release of glucocorticoids endogenously during stress, resulting in binding of the synthetic steroid to the glucocorticoid receptor and the associated inhibition of the production of proinflammatory cytokines. In this model, dexamethasone reduces inflammation by stimulating the biological mechanism that reduces inflammation following a threat such as immune challenge.

Initial support for dexamethasone as a treatment for COVID-19 came from the United Kingdom’s RECOVERY trial [62], which assigned over 6,000 hospitalized COVID-19 patients to the standard of care (SOC) or treatment (dexamethasone) arms of the trial at a 2:1 ratio. At the time of randomization, some patients were ventilated (16%), others were on non-invasive oxygen (60%), and others were breathing independently (24%). Patients in the treatment arm were administered dexamethasone either orally or intravenously at 6 mg per day for up to 10 days. The primary end-point was the patient’s status at 28-days post-randomization (mortality, discharge, or continued hospitalization), and secondary outcomes analyzed included the progression to invasive mechanical ventilation over the same period. The 28-day mortality rate was found to be lower in the treatment group than in the SOC group (21.6% vs 24.6%, *p* < 0.001). However, the effect was driven by improvements in patients receiving mechanical ventilation or supplementary oxygen. One possible confounder is that patients receiving mechanical ventilation tended to be younger than patients who were not receiving respiratory support (by 10 years on average) and to have had symptoms for a longer period. However, adjusting for age did not change the conclusions, although the duration of symptoms was found to be significantly associated with the effect of dexamethasone administration. Thus, this large, randomized, and multi-site, albeit not placebo-controlled, study suggests that administration of dexamethasone to patients who are unable to breathe independently may significantly improve survival outcomes. Additionally, dexamethasone is a widely available and affordable medication, raising the hope that it could be made available to COVID-19 patients globally.

It is not surprising that administration of an immunosuppressant would be most beneficial in severe cases where the immune system was dysregulated towards inflammation. However, it is also unsurprising that care must be taken in administering an immunosuppressant to patients fighting a viral infection. In particular, the concern has been raised that treatment with dexamethasone might increase patient susceptibility to concurrent (e.g., nosocomial) infections [293]. Additionally, the drug could potentially slow viral clearance and inhibit patients’ ability to develop antibodies to SARS-CoV-2 [59,293], with the lack of data about viral clearance being put forward as a major limitation of the RECOVERY trial [294]. Furthermore, dexamethasone has been associated with side effects that include psychosis, glucocorticoid-induced diabetes, and avascular necrosis [59], and the RECOVERY trial did not report outcomes with enough detail to be able to determine whether they observed similar complications. The effects of dexamethasone have also been found to differ among populations, especially in high-income versus middle- or low-income countries [295]. However, since the RECOVERY trial’s results were released, strategies have been proposed for administering dexamethasone alongside more targeted treatments to minimize the likelihood of negative side effects [293]. Given the available evidence, dexamethasone is currently the most promising treatment for severe COVID-19.

### 1.2 Favipiravir

The effectiveness of favipiravir for treating patients with COVID-19 is currently under investigation. Evidence for the drug inhibiting viral RNA polymerase are based on time-of-drug addition studies that found that viral loads were reduced with the addition of favipiravir in early times post-infection [80,83,84]. An open-label, nonrandomized, before-after controlled study for COVID-19 was recently conducted [296]. The study included 80 COVID-19 patients (35 treated with favipiravir, 45 control) from the isolation ward of the National Clinical Research Center for Infectious Diseases (The Third People’s Hospital of Shenzhen), Shenzhen, China. The patients in the control group were treated with other antivirals, such as lopinavir and ritonavir. It should be noted that although the control patients received antivirals, two subsequent large-scale analyses, the WHO Solidarity trial and the Randomized Evaluation of COVID-19 Therapy (RECOVERY) trial, identified no effect of lopinavir or of a lopinavir-ritonavir combination, respectively, on the metrics of COVID-19-related mortality that each assessed [93,297,298]. Treatment was applied on days 2–14; treatment stopped either when viral clearance was confirmed or at day 14. The efficacy of the treatment was measured by, first, the time until viral clearance using Kaplan-Meier survival curves, and, second, the improvement rate of chest computed tomography (CT) scans on day 14 after treatment. The study found that favipiravir increased the speed of recovery, measured as viral clearance from the patient by RT-PCR, with patients receiving favipiravir recovering in four days compared to 11 days for patients receiving antivirals such as lopinavir and ritonavir. Additionally, the lung CT scans of patients treated with favipiravir showed significantly higher improvement rates (91%) on day 14 compared to control patients (62%, *p* = 0.004). However, there were adverse side effects in 4 (11%) favipiravir-treated patients and 25 (56%) control patients. The adverse side effects included diarrhea, vomiting, nausea, rash, and liver and kidney injury. Despite the study reporting clinical improvement in favipiravir-treated patients, several study design issues are problematic and lower confidence in the overall conclusions. For example, the study was neither randomized nor blinded. Moreover, the selection of patients did not take into consideration important factors such as previous clinical conditions or sex, and there was no age categorization. Additionally, it should be noted that this study was temporarily retracted and then restored without an explanation [299].

In late 2020 and early 2021, the first randomized controlled trials of favipiravir for the treatment of COVID-19 released results [300,301,302]. The first [300] used a randomized, controlled, open-label design to compare two drugs, favipiravir and baloxavir marboxil, to SOC alone. Here, SOC included antivirals such as lopinavir/ritonavir and was administered to all patients. The primary endpoint analyzed was viral clearance at day 14. The sample size for this study was very small, with 29 total patients enrolled, and no significant effect of the treatments was found for the primary or any of the secondary outcomes analyzed, which included mortality. The second study [301] was larger, with 96 patients enrolled, and included only individuals with mild to moderate symptoms who were randomized into two groups: one receiving chloroquine (CQ) in addition to SOC, and the other receiving favipiravir in addition to SOC. This study reported a non-significant trend for patients receiving favipiravir to have a shorter hospital stay (13.29 days compared to 15.89 for CQ, *p* = 0.06) and less likelihood of progressing to mechanical ventilation (*p* = 0.118) or to an oxygen saturation < 90% (*p* = 0.129). These results, combined with the fact that favipiravir was being compared to CQ, which is now widely understood to be ineffective for treating COVID-19, thus do not suggest that favipiravir was likely to have had a strong effect on these outcomes. On the other hand, another trial of 60 patients reported a significant effect of favipiravir on viral clearance at four days (a secondary endpoint), but not at 10 days (the primary endpoint) [302]. This study, as well as a prior study of favipiravir [303], also reported that the drug was generally well-tolerated. Thus, in combination, these small studies suggest that the effects of favipiravir as a treatment for COVID-19 cannot be determined based on the available evidence, but additionally, none raise major concerns about the safety profile of the drug.

### 1.3 Remdesivir

At the outset of the COVID-19 pandemic, remdesivir did not have any have any FDA-approved use. A clinical trial in the Democratic Republic of Congo found some evidence of effectiveness against ebola virus disease (EVD), but two antibody preparations were found to be more effective, and remdesivir was not pursued [304]. Remdesivir also inhibits polymerase and replication of the coronaviruses MERS-CoV and SARS-CoV-1 in cell culture assays with submicromolar IC50s [305]. It has also been found to inhibit SARS-CoV-2, showing synergy with CQ *in vitro* [88].

Remdesivir was first used on some COVID-19 patients under compassionate use guidelines [308]. All were in late stages of COVID-19 infection, and initial reports were inconclusive about the drug’s efficacy. Gilead Sciences, the maker of remdesivir, led a recent publication that reported outcomes for compassionate use of the drug in 61 patients hospitalized with confirmed COVID-19. Here, 200 mg of remdesivir was administered intravenously on day 1, followed by a further 100 mg/day for 9 days [92]. There were significant issues with the study design, or lack thereof. There was no randomized control group. The inclusion criteria were variable: some patients only required low doses of oxygen, while others required ventilation. The study included many sites, potentially with variable inclusion criteria and treatment protocols. The patients analyzed had mixed demographics. There was a short follow-up period of investigation. Eight patients were excluded from the analysis mainly due to missing post-baseline information; thus, their health was unaccounted for. Therefore, even though the study reported clinical improvement in 68% of the 53 patients ultimately evaluated, due to the significant issues with study design, it could not be determined whether treatment with remdesivir had an effect or whether these patients would have recovered regardless of treatment. Another study comparing 5- and 10-day treatment regimens reported similar results but was also limited because of the lack of a placebo control [309]. These studies did not alter the understanding of the efficacy of remdesivir in treating COVID-19, but the encouraging results provided motivation for placebo-controlled studies.

The double-blind placebo-controlled ACTT-1 trial [89,90] recruited 1,062 patients and randomly assigned them to placebo treatment or treatment with remdesivir. Patients were stratified for randomization based on site and the severity of disease presentation at baseline [89]. The treatment was 200 mg on day 1, followed by 100 mg on days 2 through 10. Data was analyzed from a total of 1,059 patients who completed the 29-day course of the trial, with 517 assigned to remdesivir and 508 to placebo [89]. The two groups were well matched demographically and clinically at baseline. Those who received remdesivir had a median recovery time of 10 days, as compared with 15 days in those who received placebo (rate ratio for recovery, 1.29; 95% confidence interval (CI), 1.12 to 1.49; *p* < 0.001). The Kaplan-Meier estimates of mortality by 14 days were 6.7% with remdesivir and 11.9% with placebo, with a hazard ratio (HR) for death of 0.55 and a 95% CI of 0.36 to 0.83, and at day 29, remdesivir corresponded to 11.4% and the placebo to 15.2% (HR: 0.73; 95% CI, 0.52 to 1.03). Serious adverse events were reported in 131 of the 532 patients who received remdesivir (24.6%) and in 163 of the 516 patients in the placebo group (31.6%). This study also reported an association between remdesivir administration and both clinical improvement and a lack of progression to more invasive respiratory intervention in patients receiving non-invasive and invasive ventilation at randomization [89]. Largely on the results of this trial, the FDA reissued and expanded the EUA for remdesivir for the treatment of hospitalized COVID-19 patients ages twelve and older [310]. Additional clinical trials [88,311,312,313,314] are currently underway to evaluate the use of remdesivir to treat COVID-19 patients at both early and late stages of infection and in combination with other drugs (Figure 2). As of October 22, 2020, remdesivir received FDA approval based on three clinical trials [315].

However, results suggesting no effect of remdesivir on survival were reported by the WHO Solidarity trial [93]. Patients were randomized in equal proportions into four experimental conditions and a control condition, corresponding to four candidate treatments for COVID-19 and SOC, respectively; no placebo was administered. The 2,750 patients in the remdesivir group were administered 200 mg intravenously on the first day and 100 mg on each subsequent day until day 10 and assessed for in-hospital death (primary endpoint), duration of hospitalization, and progression to mechanical ventilation. There were also 2,708 control patients who would have been eligible and able to receive remdesivir were they not assigned to the control group. A total of 604 patients among these two cohorts died during initial hospitalization, with 301 in the remdesivir group and 303 in the control group. The rate ratio of death between these two groups was therefore not significant (0.95, *p* = 0.50), suggesting that the administration of remdesivir did not affect survival. The two secondary analyses similarly did not find any effect of remdesivir. Additionally, the authors compared data from their study with data from three other studies of remdesivir (including [89]) stratified by supplemental oxygen status. A meta-analysis of the four studies yielded an overall rate ratio for death of 0.91 (*p* = 0.20). These results thus do not support the previous findings that remdesivir reduced median recovery time and mortality risk in COVID-19 patients.

In response to the results of the Solidarity trial, Gilead, which manufactures remdesivir, released a statement pointing to the fact that the Solidarity trial was not placebo-controlled or double-blind and at the time of release, the statement had not been peer reviewed [316]; these sentiments have been echoed elsewhere [317]. Other critiques of this study have noted that antivirals are not typically targeted at patients with severe illness, and therefore remdesivir could be more beneficial for patients with mild rather than severe cases [298,318]. However, the publication associated with the trial sponsored by Gilead did purport an effect of remdesivir on patients with severe disease, identifying an 11 versus 18 day recovery period (rate ratio for recovery: 1.31, 95% CI 1.12 to 1.52) [89]. Additionally, a smaller analysis of 598 patients, of whom two-thirds were randomized to receive remdesivir for either 5 or 10 days, reported a small effect of treatment with remdesivir for five days relative to standard of care in patients with moderate COVID-19 [319]. These results suggest that remdesivir could improve outcomes for patients with moderate COVID-19, but that additional information would be needed to understand the effects of different durations of treatment. Therefore, the Solidarity trial may point to limitations in the generalizability of other research on remdesivir, especially since the broad international nature of the Solidarity clinical trial, which included countries with a wide range of economic profiles and a variety of healthcare systems, provides a much-needed global perspective in a pandemic [298]. On the other hand, only 62% of patients in the Solidarity trial were randomized on the day of admission or one day afterwards [93], and concerns have been raised that differences in disease progression could influence the effectiveness of remdesivir [298]. Despite the findings of the Solidarity trial, remdesivir remains available for the treatment of COVID-19 in many places. Remdesivir has also been investigated in combination with other drugs, such as baricitinib, which is an inhibitor of Janus kinase 1 and 2 [320]; the FDA has issued an EUA for the combination of remdesivir and baricitinib in adult and pediatric patients [321]. Follow-up studies are needed and, in many cases, are underway to further investigate remdesivir-related outcomes.

Similarly, the extent to which the remdesivir dosing regimen could influence outcomes continues to be under consideration. A randomized, open-label trial compared the effect of remdesivir on 397 patients with severe COVID-19 over 5 versus 10 days [91,309], complementing the study that found that a 5-day course of remdesivir improved outcomes for patients with moderate COVID-19 but a 10-day course did not [319]. Patients in the two groups were administered 200 mg of remdesivir intravenously on the first day, followed by 100 mg on the subsequent four or nine days, respectively. The two groups differed significantly in their clinical status, with patients assigned to the 10-day group having more severe illness. This study also differed from most because it included not only adults, but also pediatric patients as young as 12 years old. It reported no significant differences across several outcomes for patients receiving a 5-day or 10-day course, when correcting for baseline clinical status. The data did suggest that the 10-day course might reduce mortality in the most severe patients at day 14, but the representation of this group in the study population was too low to justify any conclusions [309]. Thus, additional research is also required to determine whether the dosage and duration of remdesivir administration influences outcomes.

In summary, remdesivir is the first FDA approved anti-viral against SARS-CoV-2 as well as the first FDA approved COVID-19 treatment. Early investigations of this drug established proof of principle that drugs targeting the virus can benefit COVID-19 patients. Moreover, one of the most successful strategies for developing therapeutics for viral diseases is to target the viral replication machinery, which are typically virally encoded polymerases. Small molecule drugs targeting viral polymerases are the backbones of treatments for other viral diseases including human immunodeficiency virus (HIV) and herpes. Notably, the HIV and herpes polymerases are a reverse transcriptase and a DNA polymerase, respectively, whereas SARS-CoV-2 encodes an RdRP, so most of the commonly used polymerase inhibitors are not likely to be active against SARS-CoV-2. In clinical use, polymerase inhibitors show short term benefits for HIV patients, but for long term benefits they must be part of combination regimens. They are typically combined with protease inhibitors, integrase inhibitors, and even other polymerase inhibitors. Remdesivir provides evidence that a related approach may be beneficial for the treatment of COVID-19.

### 1.4 Hydroxychloroquine and Chloroquine

CQ and hydroxychloroquine (HCQ) increase cellular pH by accumulating in their protonated form inside lysosomes [95,322]. This shift in pH inhibits the breakdown of proteins and peptides by the lysosomes during the process of proteolysis [95]. Interest in CQ and HCQ for treating COVID-19 was catalyzed by a mechanism observed in *in vitro* studies of both SARS-CoV-1 and SARS-CoV-2. In one study, CQ inhibited viral entry of SARS-CoV-1 into Vero E6 cells, a cell line that was derived from Vero cells in 1968, through the elevation of endosomal pH and the terminal glycosylation of ACE2 [96]. Increased pH within the cell, as discussed above, inhibits proteolysis, and terminal glycosylation of ACE2 is thought to interfere with virus-receptor binding. An *in vitro* study of SARS-CoV-2 infection of Vero cells found both HCQ and CQ to be effective in inhibiting viral replication, with HCQ being more potent [97]. Additionally, an early case study of three COVID-19 patients reported the presence of antiphospholipid antibodies in all three patients [323]. Antiphospholipid antibodies are central to the diagnosis of the antiphospholipid syndrome, a disorder that HCQ has often been used to treat [324,325,326]. Because the 90% effective concentration (EC_90_) of CQ in Vero E6 cells (6.90 μM) can be achieved in and tolerated by rheumatoid arthritis (RA) patients, it was hypothesized that it might also be possible to achieve the effective concentration in COVID-19 patients [327]. Additionally, clinical trials have reported HCQ to be effective in treating HIV [328] and chronic Hepatitis C [329]. Together, these studies triggered initial enthusiasm about the therapeutic potential for HCQ and CQ against COVID-19. HCQ/CQ has been proposed both as a treatment for COVID-19 and a prophylaxis against SARS-CoV-2 exposure, and trials often investigated these drugs in combination with azithromycin (AZ) and/or zinc supplementation. However, as more evidence has emerged, it has become clear that HCQ/CQ offer no benefits against SARS-CoV-2 or COVID-19.

#### 1.4.1 Trials Assessing Therapeutic Administration of HCQ/CQ

The initial study evaluating HCQ as a treatment for COVID-19 patients was published on March 20, 2020 by Gautret et al. [98]. This non-randomized, non-blinded, non-placebo clinical trial compared HCQ to SOC in 42 hospitalized patients in southern France. It reported that patients who received HCQ showed higher rates of virological clearance by nasopharyngeal swab on days 3–6 when compared to SOC. This study also treated six patients with both HCQ + AZ and found this combination therapy to be more effective than HCQ alone. However, the design and analyses used showed weaknesses that severely limit interpretability of results, including the small sample size and the lack of: randomization, blinding, placebo (no “placebo pill” given to SOC group), Intention-To-Treat analysis, correction for sequential multiple comparisons, and trial pre-registration. Furthermore, the trial arms were entirely confounded by the hospital and there were false negative outcome measurements (see [330]). Two of these weaknesses are due to inappropriate data analysis and can therefore be corrected *post hoc* by recalculating the p-values (lack of Intention-To-Treat analysis and multiple comparisons). However, all other weaknesses are fundamental design flaws and cannot be corrected for. Thus, the conclusions cannot be generalized outside of the study. The International Society of Antimicrobial Chemotherapy, the scientific organization that publishes the journal where the article appeared, subsequently announced that the article did not meet its expected standard for publications [99], although it has not been officially retracted.

Because of the preliminary data presented in this study, HCQ treatment was subsequently explored by other researchers. About one week later, a follow-up case study reported that 11 consecutive patients were treated with HCQ + AZ using the same dosing regimen [331]. One patient died, two were transferred to the intensive care unit (ICU), and one developed a prolonged QT interval, leading to discontinuation of HCQ + AZ administration. As in the Gautret et al. study, the outcome assessed was virological clearance at day 6 post-treatment, as measured from nasopharyngeal swabs. Of the ten living patients on day 6, eight remained positive for SARS-CoV-2 RNA. Like in the original study, interpretability was severely limited by the lack of a comparison group and the small sample size. However, these results stand in contrast to the claims by Gautret et al. that all six patients treated with HCQ + AZ tested negative for SARS-CoV-2 RNA by day 6 post-treatment. This case study illustrated the need for further investigation using robust study design to evaluate the efficacy of HCQ and/or CQ.

On April 10, 2020, a randomized, non-placebo trial of 62 COVID-19 patients at the Renmin Hospital of Wuhan University was released [332]. This study investigated whether HCQ decreased time to fever break or time to cough relief when compared to SOC [332]. This trial found HCQ decreased both average time to fever break and average time to cough relief, defined as mild or no cough. While this study improved on some of the methodological flaws in Gautret et al. by randomizing patients, it also had several flaws in trial design and data analysis that prevent generalization of the results. These weaknesses include the lack of placebo, lack of correction for multiple primary outcomes, inappropriate choice of outcomes, lack of sufficient detail to understand analysis, drastic disparities between pre-registration [333] and published protocol (including differences in the inclusion and exclusion criteria, the number of experimental groups, the number of patients enrolled, and the outcome analyzed), and small sample size. The choice of outcomes may be inappropriate as both fevers and cough may break periodically without resolution of illness. Additionally, for these outcomes, the authors reported that 23 of 62 patients did not have a fever and 25 of 62 patients did not have a cough at the start of the study, but the authors failed to describe how these patients were included in a study assessing time to fever break and time to cough relief. It is important to note here that the authors claimed “neither the research performers nor the patients were aware of the treatment assignments.” This blinding seems impossible in a non-placebo trial because at the very least, providers would know whether they were administering a medication or not, and this knowledge could lead to systematic differences in the administration of care. Correction for multiple primary outcomes can be adjusted *post hoc* by recalculating p-values, but all of the other issues were design and statistical weaknesses that cannot be corrected for. Additionally, disparities between the pre-registered and published protocols raise concerns about experimental design. The design limitations mean that the conclusions cannot be generalized outside of the study.

A second randomized trial, conducted by the Shanghai Public Health Clinical Center, analyzed whether HCQ increased rates of virological clearance at day 7 in respiratory pharyngeal swabs compared to SOC [334]. This trial was published in Chinese along with an abstract in English, and only the English abstract was read and interpreted for this review. The trial found comparable outcomes in virological clearance rate, time to virological clearance, and time to body temperature normalization between the treatment and control groups. The small sample size is one weakness, with only 30 patients enrolled and 15 in each arm. This problem suggests the study is underpowered to detect potentially useful differences and precludes interpretation of results. Additionally, because only the abstract could be read, other design and analysis issues could be present. Thus, though these studies added randomization to their assessment of HCQ, their conclusions should be interpreted very cautiously. These two studies assessed different outcomes and reached differing conclusions about the efficacy of HCQ for treating COVID-19; the designs of both studies, especially with respect to sample size, meant that no general conclusions can be made about the efficacy of the drug.

Several widely reported studies on HCQ also have issues with data integrity and/or provenance. A Letter to the Editor published in *BioScience Trends* on March 16, 2020 claimed that numerous clinical trials have shown that HCQ is superior to control treatment in inhibiting the exacerbation of COVID-19 pneumonia [335]. This letter has been cited by numerous primary literature, review articles, and media alike [336,337]. However, the letter referred to 15 pre-registration identifiers from the Chinese Clinical Trial Registry. When these identifiers are followed back to the registry, most trials claim they are not yet recruiting patients or are currently recruiting patients. For all of these 15 identifiers, no data uploads or links to publications could be located on the pre-registrations. At the very least, the lack of availability of the primary data means the claim that HCQ is efficacious against COVID-19 pneumonia cannot be verified. Similarly, a recent multinational registry analysis [338] analyzed the efficacy of CQ and HCQ with and without a macrolide, which is a class of antibiotics that includes Azithromycin, for the treatment of COVID-19. The study observed 96,032 patients split into a control and four treatment conditions (CQ with and without a macrolide; HCQ with and without a macrolide). They concluded that treatment with CQ or HCQ was associated with increased risk of *de novo* ventricular arrhythmia during hospitalization. However, this study has since been retracted by *The Lancet* due to an inability to validate the data used [339]. These studies demonstrate that increased skepticism in evaluation of the HCQ/CQ and COVID-19 literature may be warranted, possibly because of the significant attention HCQ and CQ have received as possible treatments for COVID-19 and the politicization of these drugs.

Despite the fact that the study suggesting that CQ/HCQ increased risk of ventricular arrhythmia in COVID-19 patients has now been retracted, previous studies have identified risks associated with HCQ/CQ. A patient with systemic lupus erythematosus developed a prolonged QT interval that was likely exacerbated by use of HCQ in combination with renal failure [340]. A prolonged QT interval is associated with ventricular arrhythmia [341]. Furthermore, a separate study [342] investigated the safety associated with the use of HCQ with and without macrolides between 2000 and 2020. The study involved 900,000 cases treated with HCQ and 300,000 cases treated with HCQ + AZ. The results indicated that short-term use of HCQ was not associated with additional risk, but that HCQ + AZ was associated with an enhanced risk of cardiovascular complications (such as a 15% increased risk of chest pain, calibrated HR = 1.15, 95% CI, 1.05 to 1.26) and a two-fold increased 30-day risk of cardiovascular mortality (calibrated HR = 2.19; 95% CI, 1.22 to 3.94). Therefore, whether studies utilize HCQ alone or HCQ in combination with a macrolide may be an important consideration in assessing risk. As results from initial investigations of these drug combinations have emerged, concerns about the efficacy and risks of treating COVID-19 with HCQ and CQ have led to the removal of CQ/HCQ from SOC practices in several countries [343,344]. As of May 25, 2020, WHO had suspended administration of HCQ as part of the worldwide Solidarity Trial [345], and later the final results of this large-scale trial that compared 947 patients administered HCQ to 906 controls revealed no effect on the primary outcome, mortality during hospitalization (rate ratio: 1.19; *p* = 0.23)

Additional research has emerged largely identifying HCQ/CQ to be ineffective against COVID-19 while simultaneously revealing a number of significant side effects. A randomized, open-label, non-placebo trial of 150 COVID-19 patients was conducted in parallel at 16 government-designated COVID-19 centers in China to assess the safety and efficacy of HCQ [346]. The trial compared treatment with HCQ in conjunction with SOC to SOC alone in 150 infected patients who were assigned randomly to the two groups (75 per group). The primary endpoint of the study was the negative conversion rate of SARS-CoV-2 in 28 days, and the investigators found no difference in this parameter between the groups (estimated difference between SOC plus HCQ and SOC 4.1%; 95% CI, −10.3% to 18.5%). The secondary endpoints were an amelioration of the symptoms of the disease such as axillary temperature ≤36.6°C, SpO2 >94% on room air, and disappearance of symptoms like shortness of breath, cough, and sore throat. The median time to symptom alleviation was similar across different conditions (19 days in HCQ + SOC versus 21 days in SOC, *p* = 0.97). Additionally, 30% of the patients receiving SOC+HCQ reported adverse outcomes compared to 8.8% of patients receiving only SOC, with the most common adverse outcome in the SOC+HCQ group being diarrhea (10% versus 0% in the SOC group, *p* = 0.004). However, there are several factors that limit the interpretability of this study.

Most of the enrolled patients had mild-to-moderate symptoms (98%), and the average age was 46. SOC in this study included the use of antivirals (Lopinavir-Ritonavir, Arbidol, Oseltamivir, Virazole, Entecavir, Ganciclovir, and Interferon alfa), which the authors note could influence the results. Thus, they note that an ideal SOC would need to exclude the use of antivirals, but that ceasing antiviral treatment raised ethical concerns at the time that the study was conducted. In this trial, the samples used to test for the presence of the SARS-CoV-2 virus were collected from the upper respiratory tract, and the authors indicated that the use of upper respiratory samples may have introduced false negatives (e.g., [347]). Another limitation of the study that the authors acknowledge was that the HCQ treatment began, on average, at a 16-day delay from the symptom onset. The fact that this study was open-label and lacked a placebo limits interpretation, and additional analysis is required to determine whether HCQ reduces inflammatory response. Therefore, despite some potential areas of investigation identified in *post hoc* analysis, this study cannot be interpreted as providing support for HCQ as a therapeutic against COVID-19. This study provided no support for HCQ against COVID-19, as there was no difference between the two groups in either negative seroconversion at 28 days or symptom alleviation, and in fact, more severe adverse outcomes were reported in the group receiving HCQ.

Additional evidence comes from a retrospective analysis [348] that examined data from 368 COVID-19 patients across all United States Veteran Health Administration medical centers. The study retrospectively investigated the effect of the administration of HCQ (n=97), HCQ + AZ (n=113), and no HCQ (n=158) on 368 patients. The primary outcomes assessed were death and the need for mechanical ventilation. Standard supportive care was rendered to all patients. Due to the low representation of women (N=17) in the available data, the analysis included only men, and the median age was 65 years. The rate of death was 27.8% in the HCQ-only treatment group, 22.1% in the HCQ + AZ treatment group, and 14.1% in the no-HCQ group. These data indicated a statistically significant elevation in the risk of death for the HCQ-only group compared to the no-HCQ group (adjusted HR: 2.61, *p* = 0.03), but not for the HCQ + AZ group compared to the no-HCQ group (adjusted HR: 1.14; *p* = 0.72). Further, the risk of ventilation was similar across all three groups (adjusted HR: 1.43, *p* = 0.48 (HCQ) and 0.43, *p* = 0.09 (HCQ + AZ) compared to no HCQ). The study thus showed evidence of an association between increased mortality and HCQ in this cohort of COVID-19 patients but no change in rates of mechanical ventilation among the treatment conditions. The study had a few limitations: it was not randomized, and the baseline vital signs, laboratory tests, and prescription drug use were significantly different among the three groups. All of these factors could potentially influence treatment outcome. Furthermore, the authors acknowledge that the effect of the drugs might be different in females and pediatric subjects, since these subjects were not part of the study. The reported result that HCQ + AZ is safer than HCQ contradicts the findings of the previous large-scale analysis of twenty years of records that found HCQ + AZ to be more frequently associated with cardiac arrhythmia than HCQ alone [342]; whether this discrepancy is caused by the pathology of COVID-19, is influenced by age or sex, or is a statistical artifact is not presently known.

Finally, findings from the RECOVERY trial were released on October 8, 2020. This study used a randomized, open-label design to study the effects of HCQ compared to SOC in 11,197 patients at 176 hospitals in the United Kingdom [100]. Patients were randomized into either the control group or one of the treatment arms, with twice as many patients enrolled in the control group as any treatment group. Of the patients eligible to receive HCQ, 1,561 were randomized into the HCQ arm, and 3,155 were randomized into the control arm. The demographics of the HCQ and control groups were similar in terms of average age (65 years), proportion female (approximately 38%), ethnic make-up (73% versus 76% white), and prevalence of pre-existing conditions (56% versus 57% overall). In the HCQ arm of the study, patients received 800 mg at baseline and again after 6 hours, then 400 mg at 12 hours and every subsequent 12 hours. The primary outcome analyzed was all-cause mortality, and patient vital statistics were reported by physicians upon discharge or death, or else at 28 days following HCQ administration if they remained hospitalized. The secondary outcome assessed was the combined risk of progression to invasive mechanical ventilation or death within 28 days. By the advice of an external data monitoring committee, the HCQ arm of the study was reviewed early, leading to it being closed due a lack of support for HCQ as a treatment for COVID-19. COVID-19-related mortality was not affected by HCQ in the RECOVERY trial (rate ratio, 1.09; 95% CI, 0.97 to 1.23; *p* = 0.15), but cardiac events were increased in the HCQ arm (0.4 percentage points), as was the duration of hospitalization (rate ratio for discharge alive within 28 days: 0.90; 95% CI, 0.83 to 0.98) and likelihood of progression to mechanical ventilation or death (risk ratio 1.14; 95% CI, 1.03 to 1.27). This large-scale study thus builds upon studies in the United States and China to suggest that HCQ is not an effective treatment, and in fact may negatively impact COVID-19 patients due to its side effects. Therefore, though none of the studies have been blinded, examining them together makes it clear that the available evidence points to significant dangers associated with the administration of HCQ to hospitalized COVID-19 patients, without providing any support for its efficacy.

#### 1.4.2 HCQ for the Treatment of Mild Cases

One additional possible therapeutic application of HCQ considered was the treatment of mild COVID-19 cases in otherwise healthy individuals. This possibility was assessed in a randomized, open-label, multi-center analysis conducted in Catalonia (Spain) [349]. This analysis enrolled adults 18 and older who had been experiencing mild symptoms of COVID-19 for fewer than five days. Participants were randomized into an HCQ arm (N=136) and a control arm (N=157), and those in the treatment arm were administered 800 mg of HCQ on the first day of treatment followed by 400 mg on each of the subsequent six days. The primary outcome assessed was viral clearance at days 3 and 7 following the onset of treatment, and secondary outcomes were clinical progression and time to complete resolution of symptoms. No significant differences between the two groups were found: the difference in viral load between the HCQ and control groups was 0.01 (95% CI, −0.28 to 0.29) at day 3 and −0.07 (95% CI −0.44 to 0.29) at day 7, the relative risk of hospitalization was 0.75 (95% CI, 0.32 to 1.77), and the difference in time to complete resolution of symptoms was −2 days (*p* = 0.38). This study thus suggests that HCQ does not improve recovery from COVID-19, even in otherwise healthy adult patients with mild symptoms.

#### 1.4.3 Prophylactic Administration of HCQ

An initial study of the possible prophylactic application of HCQ utilized a randomized, double-blind, placebo-controlled design to analyze the administration of HCQ prophylactically [350]. Asymptomatic adults in the United States and Canada who had been exposed to SARS-CoV-2 within the past four days were enrolled in an online study to evaluate whether administration of HCQ over five days influenced the probability of developing COVID-19 symptoms over a 14-day period. Of the participants, 414 received HCQ and 407 received a placebo. No significant difference in the rate of symptomatic illness was observed between the two groups (11.8% HCQ, 14.3% placebo, *p* = 0.35). The HCQ condition was associated with side effects, with 40.1% of patients reporting side effects compared to 16.8% in the control group (*p* < 0.001). However, likely due to the high enrollment of healthcare workers (66% of participants) and the well-known side effects associated with HCQ, a large number of participants were able to correctly identify whether they were receiving HCQ or a placebo (46.5% and 35.7%, respectively). Furthermore, due to a lack of availability of diagnostic testing, only 20 of the 107 cases were confirmed with a PCR-based test to be positive for SARS-CoV-2. The rest were categorized as “probable” or “possible” cases by a panel of four physicians who were blind to the treatment status. One possible confounder is that a patient presenting one or more symptoms, which included diarrhea, was defined as a “possible” case, but diarrhea is also a common side effect of HCQ. Additionally, four of the twenty PCR-confirmed cases did not develop symptoms until after the observation period had completed, suggesting that the 14-day trial period may not have been long enough or that some participants also encountered secondary exposure events. Finally, in addition to the young age of the participants in this study, which ranged from 32 to 51, there were possible impediments to generalization introduced by the selection process, as 2,237 patients who were eligible but had already developed symptoms by day 4 were enrolled in a separate study. It is therefore likely that asymptomatic cases were over-represented in this sample, which would not have been detected based on the diagnostic criteria used. Therefore, while this study does represent the first effort to conduct a randomized, double-blind, placebo-controlled investigation of HCQ’s effect on COVID-19 prevention after SARS-CoV-2 exposure in a large sample, the lack of PCR tests and several other design flaws significantly impede interpretation of the results. However, in line with the results from therapeutic studies, once again no evidence was found suggesting an effect of HCQ against COVID-19.

A second study [351] examined the effect of administering HCQ to healthcare workers as a pre-exposure prophylactic. The primary outcome assessed was the conversion from SARS-CoV-2 negative to SARS-CoV-2 positive status over the 8 week study period. This study was also randomized, double-blind, and placebo-controlled, and it sought to address some of the limitations of the first prophylactic study. The goal was to enroll 200 healthcare workers, preferentially those working with COVID-19 patients, at two hospitals within the University of Pennsylvania hospital system in Philadelphia, PA. Participants were randomized 1:1 to receive either 600 mg of HCQ daily or a placebo, and their SARS-CoV-2 infection status and antibody status were assessed using RT-PCR and serological testing, respectively, at baseline, 4 weeks, and 8 weeks following the beginning of the treatment period. The statistical design of the study accounted for interim analyses at 50 and 100 participants in case efficacy or futility of HCQ for prophylaxis became clear earlier than completion of enrollment. The 139 individuals enrolled comprised a study population that was fairly young (average age 33) and made of largely of people who were white, women, and without pre-existing conditions. At the second interim analysis, more individuals in the treatment group than the control group had contracted COVID-19 (4 versus 3), causing the estimated z-score to fall below the pre-established threshold for futility. As a result, the trial was terminated early, offering additional evidence against the use of HCQ for prophylaxis.

#### 1.4.4 Summary of HCQ/CQ Research Findings

Early *in vitro* evidence indicated that HCQ could be an effective therapeutic against SARS-CoV-2 and COVID-19, leading to significant media attention and public interest in its potential as both a therapeutic and prophylactic. Initially it was hypothesized that CQ/HCQ might be effective against SARS-CoV-2 in part because CQ and HCQ have both been found to inhibit the expression of CD154 in T-cells and to reduce TLR signaling that leads to the production of pro-inflammatory cytokines [352]. Clinical trials for COVID-19 have more often used HCQ rather than CQ because it offers the advantages of being cheaper and having fewer side effects than CQ. However, research has not found support for a positive effect of HCQ on COVID-19 patients. Multiple clinical studies have already been carried out to assess HCQ as a therapeutic agent for COVID-19, and many more are in progress. To date, none of these studies have used randomized, double-blind, placebo-controlled designs with a large sample size, which would be the gold standard. Despite the design limitations (which would be more likely to produce false positives than false negatives), initial optimism about HCQ has largely dissipated. The most methodologically rigorous analysis of HCQ as a prophylactic [350] found no significant differences between the treatment and control groups, and the WHO’s global Solidarity trial similarly reported no effect of HCQ on mortality [93]. Thus, HCQ/CQ are not likely to be effective therapeutic or prophylactic agents against COVID-19. One case study identified drug-induced phospholipidosis as the cause of death for a COVID-19 patient treated with HCQ [272], suggesting that in some cases, the proposed mechanism of action may ultimately be harmful. Additionally, one study identified an increased risk of mortality in older men receiving HCQ, and administration of HCQ and HCQ + AZ did not decrease the use of mechanical ventilation in these patients [348]. HCQ use for COVID-19 could also lead to shortages for anti-malarial or anti-rheumatic use, where it has documented efficacy. Despite significant early attention, these drugs appear to be ineffective against COVID-19. Several countries have now removed CQ/HCQ from their SOC for COVID-19 due to the lack of evidence of efficacy and the frequency of adverse effects.

### 1.5 ACE Inhibitors and Angiotensin II Receptor Blockers

Several clinical trials testing the effects of ACEIs or ARBs on COVID-19 outcomes are ongoing [353,354,355,356,357,358,359]. Clinical trials are needed because the findings of the various observational studies bearing on this topic cannot be interpreted as indicating a protective effect of the drug [360,361]. Two analyses [353,359] have reported no effect of continuing or discontinuing ARBs and ACEIs on patients admitted to the hospital for COVID-19. The first, known as REPLACE COVID [156], was a randomized, open-label study that enrolled patients who were admitted to the hospital for COVID-19 and were taking an ACEI at the time of admission. They enrolled 152 patients at 20 hospitals across seven countries and randomized them into two arms, continuation (n=75) and discontinuation (n=77). The primary outcome evaluated was a global rank score that integrated several dimensions of illness. The components of this global rank score, such as time to death and length of mechanical ventilation, were evaluated as secondary endpoints. This analysis reported no differences between the two groups in the primary or any of the secondary outcomes.

Similarly, a second study [157] used a randomized, open-label design to examine the effects of continuing versus discontinuing ARBs and ACEIs on patients hospitalized for mild to moderate COVID-19 at 29 hospitals in Brazil. This study enrolled 740 patients but had to exclude one trial site from all analyses due to the discovery of violations of Good Clinical Trial practice and data falsification. After this exclusion, 659 patients remained, with 334 randomized to discontinuation and 325 to continuation. In this study, the primary endpoint analyzed was the number of days that patients were alive and not hospitalized within 30 days of enrollment. The secondary outcomes included death (including in-hospital death separately), number of days hospitalized, and specific clinical outcomes such as heart failure or stroke. Once again, no significant differences were found between the two groups. Initial studies of randomized interventions therefore suggest that ACEIs and ARBs are unlikely to affect COVID-19 outcomes. These results are also consistent with findings from observational studies (summarized in [156]). Additional information about ACE2, observational studies of ACEIs and ARBs in COVID-19, and clinical trials on this topic have been summarized [362]. Therefore, despite the promising potential mechanism, initial results have not provided support for ACEIs and ARBs as therapies for COVID-19.

### 1.6 Tocilizumab

Human IL-6 is a 26-kDa glycoprotein that consists of 184 amino acids and contains two potential N-glycosylation sites and four cysteine residues. It binds to a type I cytokine receptor (IL-6Rα or glycoprotein 80) that exists in both membrane-bound (IL-6Rα) and soluble (sIL-6Rα) forms [363]. It is not the binding of IL-6 to the receptor that initiates pro- and/or anti-inflammatory signaling, but rather the binding of the complex to another subunit, known as IL-6Rβ or glycoprotein 130 (gp130) [363,364]. Unlike membrane-bound IL-6Rα, which is only found on hepatocytes and some types of leukocytes, gp130 is found on most cells [365]. When IL-6 binds to sIL-6Rα, the complex can then bind to a gp130 protein on any cell [365]. The binding of IL-6 to IL-6Rα is termed classical signaling, while its binding to sIL-6Rα is termed trans-signaling [365,366,367]. These two signaling processes are thought to play different roles in health and illness. For example, trans-signaling may play a role in the proliferation of mucosal T-helper TH2 cells associated with asthma, while an earlier step in this proliferation process may be regulated by classical signaling [365]. Similarly, IL-6 is known to play a role in Crohn’s Disease via trans-, but not classical, signaling [365]. Both classical and trans-signaling can occur through three independent pathways: the Janus-activated kinase-STAT3 pathway, the Ras/Mitogen-Activated Protein Kinases pathway and the Phosphoinositol-3 Kinase/Akt pathway [363]. These signaling pathways are involved in a variety of different functions, including cell type differentiation, immunoglobulin synthesis, and cellular survival signaling pathways, respectively [363]. The ultimate result of the IL-6 cascade is to direct transcriptional activity of various promoters of pro-inflammatory cytokines, such as IL-1, TFN, and even IL-6 itself, through the activity of NF-κB [363]. IL-6 synthesis is tightly regulated both transcriptionally and post-transcriptionally, and it has been shown that viral proteins can enhance transcription of the IL-6 gene by strengthening the DNA-binding activity between several transcription factors and IL-6 gene-cis-regulatory elements [368]. Therefore, drugs inhibiting the binding of IL-6 to IL-6Rα or sIL-6Rα are of interest for combating the hyperactive inflammatory response characteristic of cytokine release syndrome (CRS) and cytokine storm syndrome (CSS). TCZ is a humanized monoclonal antibody that binds both to the insoluble and soluble receptor of IL-6, providing de facto inhibition of the IL-6 immune cascade. Interest in TCZ as a possible treatment for COVID-19 was piqued by early evidence indicating that COVID-19 deaths may be induced by the hyperactive immune response, often referred to as CRS or CSS [170], as IL-6 plays a key role in this response [369]. The observation of elevated IL-6 in patients who died relative to those who recovered [170] could reflect an over-production of proinflammatory interleukins, suggesting that TCZ could potentially palliate some of the most severe symptoms of COVID-19 associated with increased cytokine production.

This early interest in TCZ as a possible treatment for COVID-19 was bolstered by a very small retrospective study in China that examined 20 patients with severe symptoms in early February 2020 and reported rapid improvement in symptoms following treatment with TCZ [176]. Subsequently, a number of retrospective studies have been conducted in several countries. Many studies use a retrospective, observational design, where they compare outcomes for COVID-19 patients who received TCZ to those who did not over a set period of time. For example, one of the largest retrospective, observational analyses released to date [171], consisting of 1,351 patients admitted to several care centers in Italy, compared the rates at which patients who received TCZ died or progressed to invasive medical ventilation over a 14-day period compared to patients receiving only SOC. Under this definition, SOC could include other drugs such as HCQ, azithromycin, lopinavir-ritonavir or darunavir-cobicistat, or heparin. While this study was not randomized, a subset of patients who were eligible to receive TCZ were unable to obtain it due to shortages; however, these groups were not directly compared in the analysis. After adjusting for variables such as age, sex, and SOFA (sequential organ failure assessment) score, they found that patients treated with TCZ were less likely to progress to invasive medical ventilation and/or death (adjusted HR = 0.61, CI 0.40–0.92, *p* = 0.020); analysis of death and ventilation separately suggests that this effect may have been driven by differences in the death rate (20% of control versus 7% of TCZ-treated patients). The study reported particular benefits for patients whose PaO_2_/FiO_2_ ratio, also known as the Horowitz Index for Lung Function, fell below a 150 mm Hg threshold. They found no differences between groups administered subcutaneous versus intravenous TCZ.

Another retrospective observational analysis of interest examined the charts of patients at a hospital in Connecticut, USA where 64% of all 239 COVID-19 patients in the study period were administered TCZ based on assignment by a standardized algorithm [172]. They found that TCZ administration was associated with more similar rates of survivorship in patients with severe versus nonsevere COVID-19 at intake, defined based on the amount of supplemental oxygen needed. They therefore proposed that their algorithm was able to identify patients presenting with or likely to develop CRS as good candidates for TCZ. This study also reported higher survivorship in Black and Hispanic patients compared to white patients when adjusted for age. The major limitation with interpretation for these studies is that there may be clinical characteristics that influenced medical practitioners decisions to administer TCZ to some patients and not others. One interesting example therefore comes from an analysis of patients at a single hospital in Brescia, Italy, where TCZ was not available for a period of time [173]. This study compared COVID-19 patients admitted to the hospital before and after March 13, 2020, when the hospital received TCZ. Therefore, patients who would have been eligible for TCZ prior to this arbitrary date did not receive it as treatment, making this retrospective analysis something of a natural experiment. Despite this design, demographic factors did not appear to be consistent between the two groups, and the average age of the control group was older than the TCZ group. The control group also had a higher percentage of males and a higher incidence of comorbidities such as diabetes and heart disease. All the same, the multivariate HR, which adjusted for these clinical and demographic factors, found a significant difference between survival in the two groups (HR=0.035, CI=0.004–0.347, *p* = 0.004). The study reported improvement of survival outcomes after the addition of TCZ to the SOC regime, with 11 of 23 patients (47.8%) admitted prior to March 13th dying compared to 2 of 62 (3.2%) admitted afterwards (HR=0.035; 95% CI, 0.004 to 0.347; *p* = 0.004). They also reported a reduced progression to mechanical ventilation in the TCZ group. However, this study also holds a significant limitation: the time delay between the two groups means that knowledge about how to treat the disease likely improved over this timeframe as well. All the same, the results of these observational retrospective studies provide support for TCZ as a pharmaceutical of interest for follow-up in clinical trials.

Other retrospective analyses have utilized a case-control design to match pairs of patients with similar baseline characteristics, only one of whom received TCZ for COVID-19. In one such study, TCZ was significantly associated with a reduced risk of progression to intensive care unit (ICU) admission or death [174]. This study examined only 20 patients treated with TCZ (all but one of the patients treated with TCZ in the hospital during the study period) and compared them to 25 patients receiving SOC. For the combined primary endpoint of death and/or ICU admission, only 25% of patients receiving TCZ progressed to an endpoint compared to 72% in the SOC group (*p* = 0.002, presumably based on a chi-square test based on the information provided in the text). When the two endpoints were examined separately, progression to invasive medical ventilation remained significant (32% SOC compared to 0% TCZ, *p* = 0.006) but not for mortality (48% SOC compared to 25% TCZ, *p* = 0.066). In contrast, a study that compared 96 patients treated with TCZ to 97 patients treated with SOC only in New York City found that differences in mortality did not differ between the two groups, but that this difference did become significant when intubated patients were excluded from the analysis [175]. Taken together, these findings suggest that future clinical trials of TCZ may want to include intubation as an endpoint. However, these studies should be approached with caution, not only because of the small number of patients enrolled and the retrospective design, but also because they performed a large number of statistical tests and did not account for multiple hypothesis testing. In general, caution must be exercised when interpreting subgroup analyses after a primary combined endpoint analysis. These last findings highlight the need to search for a balance between impairing a harmful immune response, such as the one generated during CRS/CSS, and preventing the worsening of the clinical picture of the patients by potential new viral infections. Early meta-analyses and systematic reviews have investigated the available data about TCZ for COVID-19. One meta-analysis [370] evaluated 19 studies published or released as preprints prior to July 1, 2020 and found that the overall trends were supportive of the frequent conclusion that TCZ does improve survivorship, with a significant HR of 0.41 (*p* < 0.001). This trend improved when they excluded studies that administered a steroid alongside TCZ, with a significant HR of 0.04 (*p* < 0.001). They also found some evidence for reduced invasive ventilation or ICU admission, but only when excluding all studies except a small number whose estimates were adjusted for the possible bias introduced by the challenges of stringency during the enrollment process. A systematic analysis of sixteen case-control studies of TCZ estimated an odds ratio of mortality of 0.453 (95% CI 0.376–0.547, *p* < 0.001), suggesting possible benefits associated with TCZ treatment [371]. Although these estimates are similar, it is important to note that they are drawing from the same literature and are therefore likely to be affected by the same potential biases in publication. A different systematic review of studies investigating TCZ treatment for COVID-19 analyzed 31 studies that had been published or released as pre-prints and reported that none carried a low risk of bias [372]. Therefore, the present evidence is not likely to be sufficient for conclusions about the efficacy of TCZ.

On February 11, 2021, a preprint describing the first randomized control trial of TCZ was released as part of the RECOVERY trial [177]. Of the 21,550 patients enrolled in the RECOVERY trial at the time, 4,116 adults hospitalized with COVID-19 across the 131 sites in the United Kingdom were assigned to the arm of the trial evaluating the effect of TCZ. Among them, 2,022 were randomized to receive TCZ and 2,094 were randomized to SOC, with 79% of patients in each group available for analysis at the time that the initial report was released. The primary outcome measured was 28-day mortality, and TCZ was found to reduce 28-day mortality from 33% of patients receiving SOC alone to 29% of those receiving TCZ, corresponding to a rate ratio of 0.86 (95% CI 0.77–0.96; *p* = 0.007). TCZ was also significantly associated with the probability of hospital discharge within 28 days for living patients, which was 47% in the SOC group and 54% in the TCZ group (rate ratio 1.22, 95% CI 1.12–1.34, *p* < 0.0001). A potential statistical interaction between TCZ and corticosteroids was observed, with the combination providing greater mortality benefits than TCZ alone, but the authors note that caution is advisable in light of the number of statistical tests conducted. Combining the RECOVERY trial data with data from seven smaller randomized control trials indicates that TCZ is associated with a 13% reduction in 28-day mortality (rate ratio 0.87, 95% CI 0.79–0.96, *p* = 0.005) [177].

There are possible risks associated with the administration of TCZ for COVID-19. TCZ has been used for over a decade to treat RA [373], and a recent study found the drug to be safe for pregnant and breastfeeding women [374]. However, TCZ may increase the risk of developing infections [373], and RA patients with chronic hepatitis B infections had a high risk of hepatitis B virus reactivation when TCZ was administered in combination with other RA drugs [375]. As a result, TCZ is contraindicated in patients with active infections such as tuberculosis [376]. Previous studies have investigated, with varying results, a possible increased risk of infection in RA patients administered TCZ [377,378], although another study reported that the incidence rate of infections was higher in clinical practice RA patients treated with TCZ than in the rates reported by clinical trials [379]. In the investigation of 544 Italian COVID-19 patients, the group treated with TCZ was found to be more likely to develop secondary infections, with 24% compared to 4% in the control group (*p* < 0.0001) [171]. Reactivation of hepatitis B and herpes simplex virus 1 was also reported in a small number of patients in this study, all of whom were receiving TCZ. A July 2020 case report described negative outcomes of two COVID-19 patients after receiving TCZ, including one death; however, both patients were intubated and had entered septic shock prior to receiving TCZ [380], likely indicating a severe level of cytokine production. Additionally, D-dimer and sIL2R levels were reported by one study to increase in patients treated with TCZ, which raised concerns because of the potential association between elevated D-dimer levels and thrombosis and between sIL2R and diseases where T-cell regulation is compromised [172]. An increased risk of bacterial infection was also identified in a systematic review of the literature, based on the unadjusted estimates reported [370]. In the RECOVERY trial, however, only three out of 2,022 participants in the group receiving TCZ developed adverse reactions determined to be associated with the intervention, and no excess deaths were reported [177]. TCZ administration to COVID-19 patients is not without risks and may introduce additional risk of developing secondary infections; however, while caution may be prudent when treating patients who have latent viral infections, the results of the RECOVERY trial indicate that adverse reactions to TCZ are very rare among COVID-19 patients broadly.

In summary, approximately 33% of hospitalized COVID-19 patients develop ARDS [381], which is caused by an excessive early response of the immune system which can be a component of CRS/CSS [172,376]. This overwhelming inflammation is triggered by IL-6. TCZ is an inhibitor of IL-6 and therefore may neutralize the inflammatory pathway that leads to the cytokine storm. The mechanism suggests TCZ could be beneficial for the treatment of COVID-19 patients experiencing excessive immune activity, and the RECOVERY trial reported a reduction in 28-day mortality. Interest in TCZ as a treatment for COVID-19 was also supported by two meta-analyses [370,382], but a third meta-analysis found that all of the available literature at that time carried a risk of bias [372]. Additionally, different studies used different dosages, number of doses, and methods of administration. Ongoing research may be needed to optimize administration of TCZ [383], although similar results were reported by one study for intravenous and subcutaneous administration [171]. Clinical trials that are in progress are likely to provide additional insight into the effectiveness of this drug for the treatment of COVID-19 along with how it should be administered.

### 1.7 Interferons

IFNs are a family of cytokines critical to activating the innate immune response against viral infections. Interferons are classified into three categories based on their receptor specificity: types I, II and III [369]. Specifically, IFNs I (IFN-*α* and *β*) and II (IFN-*γ*) induce the expression of antiviral proteins [384]. Among these IFNs, IFN-*β* has already been found to strongly inhibit the replication of other coronaviruses, such as SARS-CoV-1, in cell culture, while IFN *α* and *γ*were shown to be less effective in this context [384]. There is evidence that patients with higher susceptibility to ARDS indeed show deficiency in IFN-*β*. For instance, infection with other coronaviruses impairs IFN-*β* expression and synthesis, allowing the virus to escape the innate immune response [385]. On March 18 2020, Synairgen plc received approval to start a phase II trial for SNG001, an IFN-*β*-1a formulation to be delivered to the lungs via inhalation [184]. SNG001, which contains recombinant interferon beta-1a, was previously shown to be effective in reducing viral load in an *in vivo* model of swine flu and *in vitro* models of other coronavirus infections [386]. In July 2020, a press release from Synairgen stated that SNG001 reduced progression to ventilation in a double-blind, placebo-controlled, multi-center study of 101 patients with an average age in the late 50s [185]. These results were subsequently published in November 2020 [186]. The study reports that the participants were assigned at a ratio of 1:1 to receive either SNG001 or a placebo that lacked the active compound, by inhalation for up to 14 days. The primary outcome they assessed was the change in patients’ score on the WHO Ordinal Scale for Clinical Improvement (OSCI) at trial day 15 or 16. SNG001 was associated with an odds ratio of improvement on the OSCI scale of 2.32 (95% CI 1.07 – 5.04, *p* = 0.033) in the intention-to-treat analysis and 2.80 (95% CI 1.21 – 6.52, *p* = 0.017) in the per-protocol analysis, corresponding to significant improvement in the SNG001 group on the OSCI at day 15/16. Some of the secondary endpoints analyzed also showed differences: at day 28, the OR for clinical improvement on the OSCI was 3.15 (95% CI 1.39 – 7.14, *p* = 0.006), and the odds of recovery at day 15/16 and at day 28 were also significant between the two groups. Thus, this study suggested that IFN-*β*1 administered via SNG001 may improve clinical outcomes.

In contrast, the WHO Solidarity trial reported no significant effect of IFN-β−1a on patient survival during hospitalization [93]. Here, the primary outcome analyzed was in-hospital mortality, and the rate ratio for the two groups was 1.16 (95% CI, 0.96 to 1.39; *p* = 0.11) administering IFN-β−1a to 2050 patients and comparing their response to 2,050 controls. However, there are a few reasons that the different findings of the two trials might not speak to the underlying efficacy of this treatment strategy. One important consideration is the stage of COVID-19 infection analyzed in each study. The Synairgen trial enrolled only patients who were not receiving invasive ventilation, corresponding to a less severe stage of disease than many patients enrolled in the SOLIDARITY trial, as well as a lower overall rate of mortality [387]. Additionally, the methods of administration differed between the two trials, with the SOLIDARITY trial administering IFN-β−1a subcutaneously [387]. The differences in findings between the studies suggests that the method of administration might be relevant to outcomes, with nebulized IFN-β−1a more directly targeting receptors in the lungs. A trial that analyzed the effect of subcutaneously administered IFN-β−1a on patients with ARDS between 2015 and 2017 had also reported no effect on 28-day mortality [388], while a smaller study analyzing the effect of subcutaneous IFN administration did find a significant improvement in 28-day mortality for COVID-19 [389]. At present, several ongoing clinical trials are investigating the potential effects of IFN-β−1a, including in combination with therapeutics such as remdesivir [390] and administered via inhalation [184]. Thus, as additional information becomes available, a more detailed understanding of whether and under which circumstances IFN-β−1a is beneficial to COVID-19 patients should develop.

### 1.8 Potential Avenues of Interest for Therapeutic Development

Given what is currently known about these therapeutics for COVID-19, a number of related therapies beyond those explored above may also prove to be of interest. For example, the demonstrated benefit of dexamethasone and the ongoing potential of tocilizumab for treatment of COVID-19 suggests that other anti-inflammatory agents might also hold value for the treatment of COVID-19. Current evidence supporting the treatment of severe COVID-19 with dexamethasone suggests that the need to curtail the cytokine storm inflammatory response transcends the risks of immunosuppression, and other anti-inflammatory agents may therefore benefit patients in this phase of the disease. While dexamethasone is considered widely available and generally affordable, the high costs of biologics such as tocilizumab therapy may present obstacles to wide-scale distribution of this drug if it proves of value. At the doses used for RA patients, the cost for tocilizumab ranges from $179.20 to $896 per dose for the IV form and $355 for the pre-filled syringe [391]. Several other anti-inflammatory agents used for the treatment of autoimmune diseases may also be able to counter the effects of the cytokine storm induced by the virus, and some of these, such as cyclosporine, are likely to be more cost-effective and readily available than biologics [392]. While tocilizumab targets IL-6, several other inflammatory markers could be potential targets, including TNF-α. Inhibition of TNF-α by a compound such as Etanercept was previously suggested for treatment of SARS-CoV-1 [393] and may be relevant for SARS-CoV-2 as well. Another anti-IL-6 antibody, sarilumab, is also being investigated [394,395]. Baricitinib and other small molecule inhibitors of the Janus-activated kinase pathway also curtail the inflammatory response and have been suggested as potential options for SARS-CoV-2 infections [396]. Baricitinib, in particular, may be able to reduce the ability of SARS-CoV-2 to infect lung cells [397]. Clinical trials studying baricitinib in COVID-19 have already begun in the US and in Italy [398,399]. Identification and targeting of further inflammatory markers that are relevant in SARS-CoV-2 infection may be of value for curtailing the inflammatory response and lung damage.

In addition to immunosuppressive treatments, which are most beneficial late in disease progression, much research is focused on identifying therapeutics for early-stage patients. For example, although studies of HCQ have not supported the early theory-driven interest in this antiviral treatment, alternative compounds with related mechanisms may still have potential. Hydroxyferroquine derivatives of HCQ have been described as a class of bioorganometallic compounds that exert antiviral effects with some selectivity for SARS-CoV-1 *in vitro* [400]. Future work could explore whether such compounds exert antiviral effects against SARS-CoV-2 and whether they would be safer for use in COVID-19.

Another potential approach is the development of antivirals, which could be broad-spectrum, specific to coronaviruses, or targeted to SARS-CoV-2. Development of new antivirals is complicated by the fact that none have yet been approved for human coronaviruses. Intriguing new options are emerging, however. Beta-D-N4-hydroxycytidine is an orally bioavailable ribonucleotide analog showing broad-spectrum activity against RNA viruses, which may inhibit SARS-CoV-2 replication *in vitro* and *in vivo* in mouse models of HCoVs [401]. A range of other antivirals are also in development. Development of antivirals will be further facilitated as research reveals more information about the interaction of SARS-CoV-2 with the host cell and host cell genome, mechanisms of viral replication, mechanisms of viral assembly, and mechanisms of viral release to other cells; this can allow researchers to target specific stages and structures of the viral life cycle. Finally, antibodies against viruses, also known as antiviral monoclonal antibodies, could be an alternative as well and are described in detail in an above section. The goal of antiviral antibodies is to neutralize viruses through either cell-killing activity or blocking of viral replication [402]. They may also engage the host immune response, encouraging the immune system to hone in on the virus. Given the cytokine storm that results from immune system activation in response to the virus, which has been implicated in worsening of the disease, a neutralizing antibody (nAb) may be preferable. Upcoming work may explore the specificity of nAbs for their target, mechanisms by which the nAbs impede the virus, and improvements to antibody structure that may enhance the ability of the antibody to block viral activity.

Some research is also investigating potential therapeutics and prophylactics that would interact with components of the innate immune response. For example, TLRs are pattern recognition receptors that recognize pathogen- and damage-associated molecular patterns and contribute to innate immune recognition and, more generally, promotion of both the innate and adaptive immune responses [403]. In mouse models, poly(I:C) and CpG, which are agonists of Toll-like receptors TLR3 and TLR9, respectively, showed protective effects when administered prior to SARS-CoV-1 infection [404]. Therefore, TLR agonists hold some potential for broad-spectrum prophylaxis.
